# Rescuing the aberrant sex development of H3K9 demethylase Jmjd1a-deficient mice by modulating H3K9 methylation balance

**DOI:** 10.1371/journal.pgen.1007034

**Published:** 2017-09-26

**Authors:** Shunsuke Kuroki, Naoki Okashita, Shoko Baba, Ryo Maeda, Shingo Miyawaki, Masashi Yano, Miyoko Yamaguchi, Satsuki Kitano, Hitoshi Miyachi, Akihiro Itoh, Minoru Yoshida, Makoto Tachibana

**Affiliations:** 1 Division of Epigenome Dynamics, Institute of Advanced Medical Sciences, Tokushima University, Tokushima, Japan; 2 Experimental Research Center for Infectious Diseases, Institute for Virus Research, Kyoto University, Sakyo-ku, Kyoto, Japan; 3 Chemical Genomics Research Group, RIKEN Center for Sustainable Resource Science, Wako, Saitama, Japan; MRC Human Genetics Unit, UNITED KINGDOM

## Abstract

Histone H3 lysine 9 (H3K9) methylation is a hallmark of heterochromatin. H3K9 demethylation is crucial in mouse sex determination; The H3K9 demethylase Jmjd1a deficiency leads to increased H3K9 methylation at the *Sry* locus in embryonic gonads, thereby compromising *Sry* expression and causing male-to-female sex reversal. We hypothesized that the H3K9 methylation level at the *Sry* locus is finely tuned by the balance in activities between the H3K9 demethylase Jmjd1a and an unidentified H3K9 methyltransferase to ensure correct *Sry* expression. Here we identified the GLP/G9a H3K9 methyltransferase complex as the enzyme catalyzing H3K9 methylation at the *Sry* locus. Based on this finding, we tried to rescue the sex-reversal phenotype of Jmjd1a-deficient mice by modulating GLP/G9a complex activity. A heterozygous *GLP* mutation rescued the sex-reversal phenotype of Jmjd1a-deficient mice by restoring *Sry* expression. The administration of a chemical inhibitor of GLP/G9a enzyme into Jmjd1a-deficient embryos also successfully rescued sex reversal. Our study not only reveals the molecular mechanism underlying the tuning of *Sry* expression but also provides proof on the principle of therapeutic strategies based on the pharmacological modulation of epigenetic balance.

## Author summary

In eukaryotes, DNA wraps an octamer of the core histones. Covalent modifications on the histones have diverse biological functions including transcriptional regulation. Histone H3 lysine 9 (H3K9) methylation is a hallmark of transcriptionally silenced chromatin. In mammals, the sex-determining gene *Sry* initiates testis differentiation in embryonic gonads. *Sry* expression in gonads is fine-tuned in both space and time. Here, we demonstrated that fine-tuning of *Sry* expression is achieved by the balance in activities between H3K9 demethylase and H3K9 methyltransferase. We found that the GLP/G9a complex is the enzyme catalyzing H3K9 methylation of *Sry*. Based on this finding, we tried to rescue the sex-reversal phenotype of the mutant mice by modulating the H3K9 methylation balance of *Sry*. We succeeded by modulating the H3K9 methylation balance not only with a genetic approach but also with a chemical approach using an inhibitor of GLP/G9a enzyme. Aberrant histone methylation levels are associated with diseases, including cancer, and intellectual disability. Our study provides proof for the principle of therapeutic strategies based on the pharmacological modulation of histone methylation balance.

## Introduction

Covalent modifications of histone tails are epigenetic marks that play roles in many nuclear processes. Among them, methylation of histone H3 lysine 9 (H3K9) is a hallmark of transcriptionally silenced heterochromatin. Various types of H3K9 methyltransferases (“writers”) and demethylases (“erasers”) have been discovered in mammals. Considering that these H3K9 methylation “writers” and “erasers” are expressed in a cell-type-specific and developmental-stage-specific manner, H3K9 methylation levels are regulated not statically but dynamically during development [1]. In this situation, a specific combination of H3K9 methylation “writer” and “eraser” may antagonistically tune the expression levels of their target genes.

We previously demonstrated that H3K9 demethylation plays an indispensable role in mouse sex development [2]. XY mice lacking Jmjd1a (also called Kdm3a), an “eraser” for H3K9 methylation, showed male-to-female sex reversal. Jmjd1a demethylates H3K9 of the sex-determining gene *Sry* in sexually undifferentiated gonads at embryonic day 11.5 (E11.5), thereby activating *Sry* transcription. Jmjd1a deficiency induced a decrease, but not a delay of *Sry* expression in the developing gonads. We found a significant increase of dimethylated H3K9 (H3K9me2) at the *Sry* locus in embryonic gonads at E11.5 [2], suggesting the existence of an H3K9me2 “writer” that catalyzes H3K9 methylation at the *Sry* locus.

Several lines of evidence suggest that aberrant histone methylation levels are associated with diseases, including cancer, and intellectual disability [1]. Therefore, normalizing histone modification levels by manipulating the activity of the corresponding modifier is proposed as a potentially powerful therapeutic strategy [3]. Therefore, we speculated that manipulation of the activity of the H3K9me2 “writer” responsible for H3K9 methylation at the *Sry* locus normalizes *Sry* expression in the mice lacking the H3K9me2 “eraser” Jmjd1a.

In this study, we identified the H3K9 methyltransferase GLP/G9a complex as the enzyme responsible for H3K9 methylation at the *Sry* locus. Based on this finding, we aimed to rescue the aberrant sex development of Jmjd1a-deficient mice by modulating the activity of the GLP/G9a complex. The *GLP* heterozygous mutation rescued not only H3K9 hypermethylation at the *Sry* locus but also the perturbed *Sry* expression in Jmjd1a-deficient embryos. Strikingly, the sex-reversal phenotype in Jmjd1a-deficient mice was completely rescued by the *GLP* heterozygous mutation. We also aimed to rescue the phenotype by artificially manipulating the activity of the GLP/G9a complex. The administration of the GLP/G9a complex inhibitor into Jmjd1a-deficient embryos at a specific developmental time point rescued the aberrant sex differentiation of these mice. Our studies provide a novel strategy by which diseases attributed to the dysfunction of an epigenetic “eraser” can be rescued by blocking the activity of the corresponding epigenetic “writer.”

## Results

### Co-expression of GLP/G9a H3K9 complex with Jmjd1a in *Sry*-expressing pre-Sertoli cells

**Fig 1:**
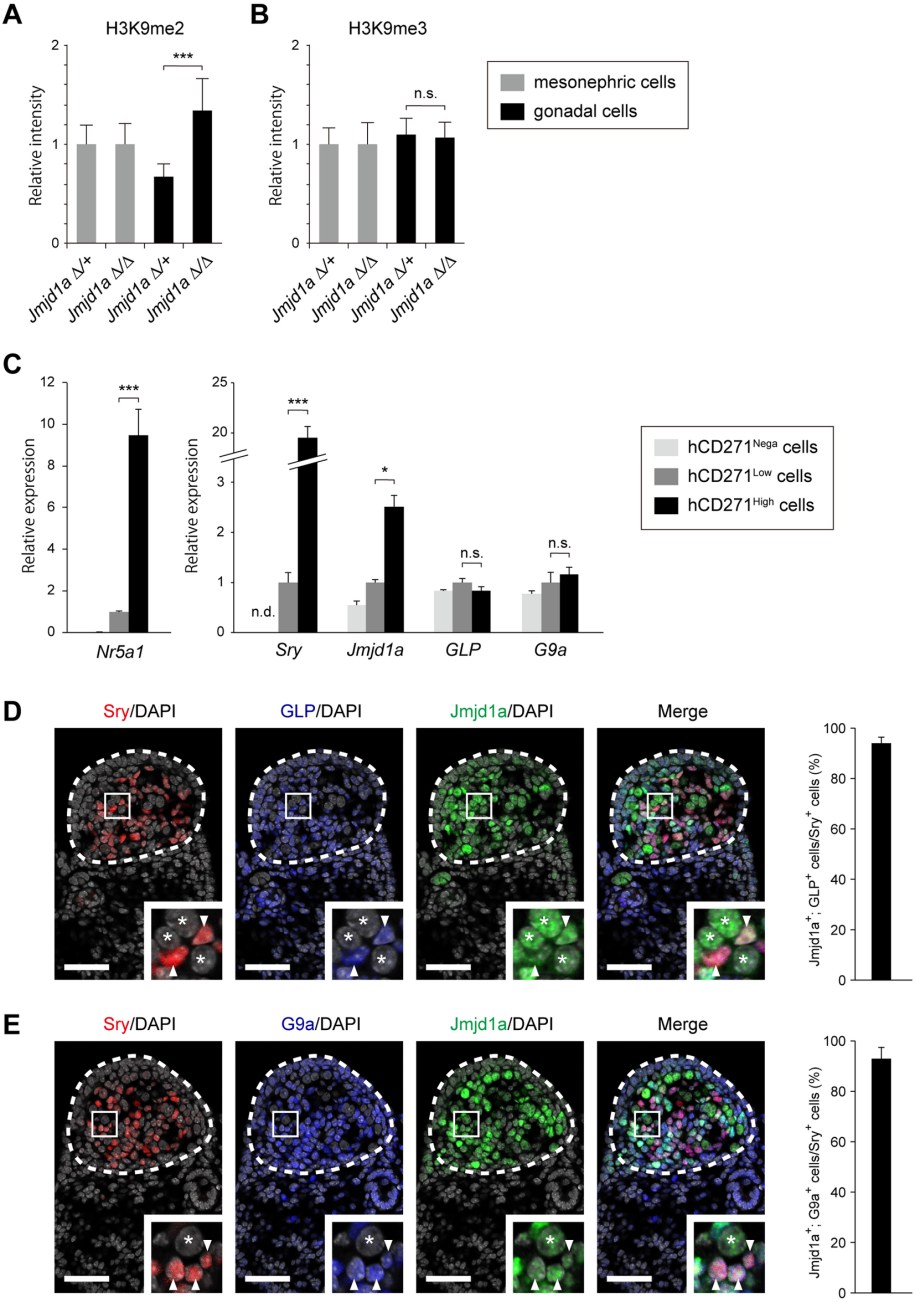
Expression of GLP/G9a H3K9 methyltransferase complex in XY embryonic gonads at E11.5. (A, B) Quantitative comparison of the immunofluorescence intensities of H3K9me2 (A) and H3K9me3 (B). Representative staining profiles are shown in S1 Fig. The intensities of H3K9 methylation of *Jmjd1a*^Δ/+^ mesonephric cells were defined as 1. Data are presented as mean ± SD. *** *P* < 0.001; n.s., not significant. (C) Relative mRNA expression profiles of *Jmjd1a*, *GLP*, and *G9a* in gonadal somatic cell populations. Gonadal somatic cells were prepared from dissociated gonads from E11.5 *Nr5a1*-*hCD271*-tg embryos and fractionated according to the expression levels of hCD271 by FACS (S2 Fig). Each fraction was subjected to mRNA expression analysis by RT-qPCR. The endogenous *Nr5a1* expression level was strictly correlated with the expression levels of hCD271 (left). *Sry* and *Jmjd1a* transcripts were substantially enriched in the hCD271-high population whereas *GLP*/*G9a* transcripts were detected in each population (right). mRNA expression levels in the hCD271-low population were defined as 1. Data are presented as mean ± SD. * *P* < 0.05, *** *P* < 0.001; n.s., not significant. (D, E) Triple immunofluorescence analyses for GLP (D) and G9a (E), counterstained with anti-Jmjd1a and anti-Sry in the center regions of E11.5 gonads. Enlarged boxes indicate co-expression of GLP (D) and G9a (E) with Jmjd1a in Sry-expressing pre-Sertoli cells (arrowheads). Asterisks represent germ cells. The population of the cells containing both signals of GLP (or G9a) and Jmjd1a among the Sry-expressing pre-Sertoli cells is presented at right. More than 200 cells per embryo (n = 3) were examined. Data are presented as mean ± SD. Scale bar, 50 μm.

In mammals, a number of enzymes possess intrinsic H3K9 methyltransferase activities, such as Suv39h1 [4], Suv39h2 [5], Eset [6], G9a [7], and GLP [8]. Among them, G9a (also called Ehmt2/Kmt1c) and GLP (also called Ehmt1/Kmt1d) form a stoichiometric heterodimer complex [9–11]. Jmjd1a deficiency resulted in the increase of H3K9me2, but not trimethylated H3K9me3 in the developing gonads at E11.5, suggesting that the enzyme counteracting Jmjd1a-mediated H3K9 demethylation produces H3K9me2 (Fig 1A and 1B, S1 Fig). We previously demonstrated that the global level of H3K9me2 in developing mouse embryos was dominantly catalyzed by the GLP/G9a complex [10]. Taking these findings together, the GLP/G9a complex was the strongest candidate for an enzyme that counteracts Jmjd1a-mediated H3K9 demethylation in the developing gonads.

Somatic cells of E11.5 embryonic gonads contain subpopulations with high and low expression levels of an orphan nuclear receptor, Nr5a1 (also called Sf-1/Ad4BP) [12]. Because a previous study demonstrated that the Nr5a1-high population contains *Sry*-expressing pre-Sertoli cells [13], we examined mRNA expression levels of *GLP*/*G9a* in this population (Fig 1C). We had established a transgenic mouse line *Nr5a1*-*hCD271*-tg, in which the human cell surface marker *CD271* (also called *LNGFR*) is expressed depending on the *Nr5a1* promoter [2] [14]. We prepared a single cell suspension from the gonads/mesonephroi of E11.5 *Nr5a1*-*hCD271*-tg embryos and then fractionated it according to the expression level of hCD271 by fluorescence-activated cell sorting (FACS) (S2 Fig). The hCD271-negative population contained mesonephric cells and germ cells [2]. As expected, quantitative RT-PCR (RT-qPCR) analysis demonstrated that the endogenous *Nr5a1* expression levels were high and low in hCD271-high and -low populations, respectively (Fig 1C, left). Concordant with the previous study [13], *Sry* transcript was substantially enriched in the hCD271-high population (Fig 1C, right). *Jmjd1a* transcript was also significantly enriched in the hCD271-high population. *GLP* and *G9a* transcripts were detected in all populations at similar levels, suggesting the ubiquitous expression of GLP/G9a complex in the developing gonads (Fig 1C, right). To address whether GLP/G9a complex and Jmjd1a were co-expressed in Sry-expressing pre-Sertoli cells, we performed triple immunostaining analyses of E11.5 embryonic gonads with antibodies against GLP/G9a, Jmjd1a, and Sry. As shown in Fig 1D and 1E, GLP/G9a complex was expressed in Sry-expressing pre-Sertoli cells. Furthermore, Sry- and GLP/G9a complex-positive cells contained robust signals for Jmjd1a (Fig 1D and 1E). Cells containing both signals of GLP (or G9a) and Jmjd1a among the Sry-expressing pre-Sertoli cells ware calculated. As summarized in Fig 1D and 1E (right panels), almost all Sry-positive cells contained the signals of both GLP/G9a complex and Jmjd1a. We therefore concluded that GLP/G9a H3K9 complex and Jmjd1a are actually co-expressed in Sry-expressing pre-Sertoli cells.

### GLP/G9a complex-mediated H3K9 methylation counteracts Jmjd1a-mediated H3K9 demethylation in gonadal somatic cells

**Fig 2:**
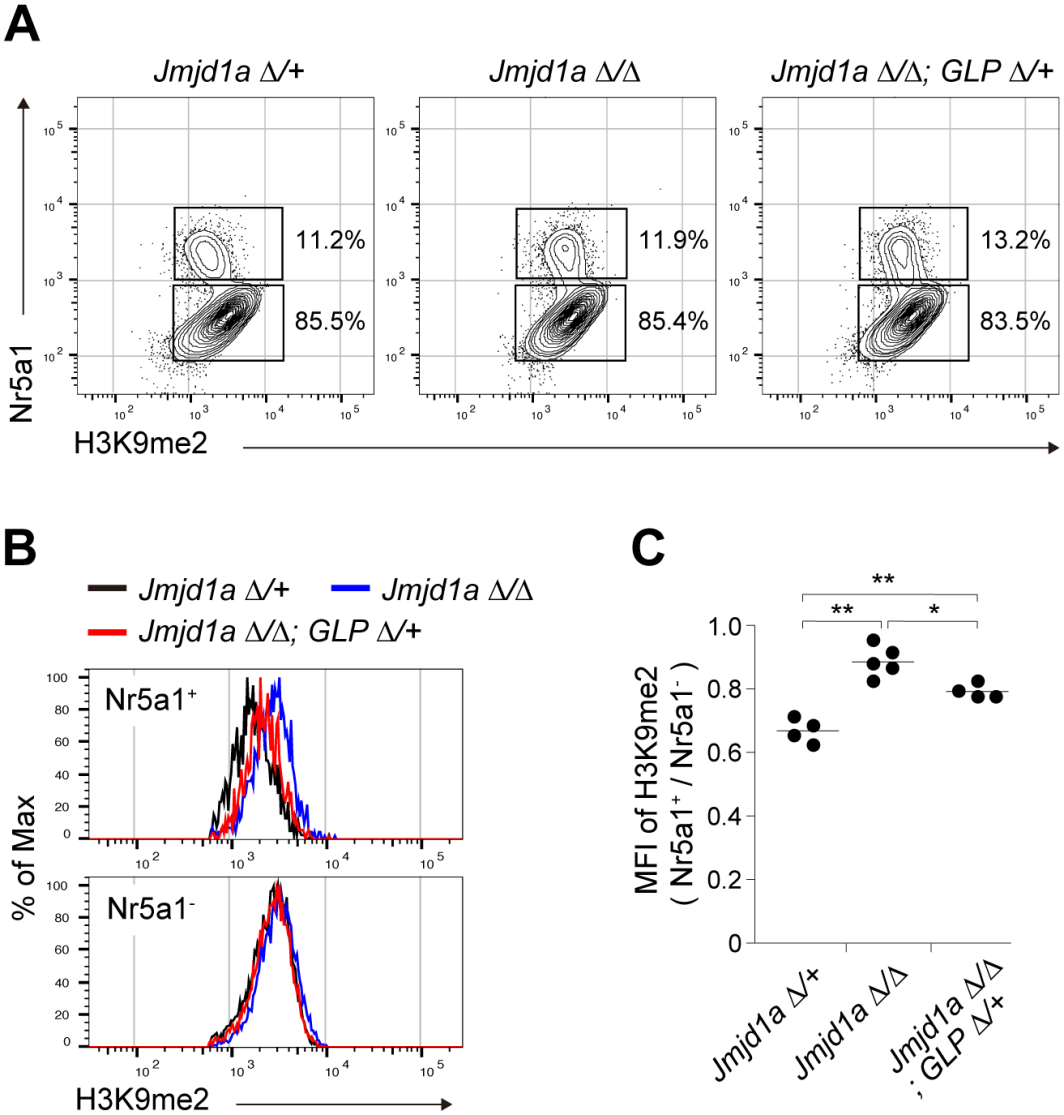
GLP/G9a complex-mediated H3K9 methylation counteracts Jmjd1a-mediated H3K9 demethylation in gonadal somatic cells. (A) Flow cytometric analyses of E11.5 gonadal somatic cells for quantifying the H3K9 methylation levels. Cells were prepared from gonads with mesonephroi and then co-stained with antibodies against H3K9me2 and Nr5a1. Upper and lower boxes indicate the populations of Nr5a1-positive gonadal somatic cells and Nr5a1-negative mesonephric cells, respectively. (B) Representative histogram analyses for H3K9me2 levels of Nr5a1-positive gonadal somatic cells (upper) and Nr5a1-negative mesonephric cells (lower). (C) Statistical analysis of H3K9me2 levels of gonadal somatic cells of the indicated genotypes. Median fluorescence intensity (MFI) values for H3K9me2 of Nr5a1-positive gonadal somatic cells were normalized to those of Nr5a1-negative cells, and then plotted (*n* = 4–5). * *P* < 0.05; ** *P* < 0.01.

We previously demonstrated that GLP is a limiting factor that controls the stability of the GLP/G9a heterodimer complex. In addition, the heterodimer formation of GLP/G9a was shown to be essential for H3K9 methylation *in vivo* [10] [15]. Thus, we first examined whether a *GLP* mutation might rescue the increased H3K9me2 levels in Jmjd1a-deficient mice. A homozygous *GLP* mutation in mice leads to embryonic lethality around E9.5 [10]. We therefore generated mice heterozygous for the *GLP* mutation (*GLP*^Δ^; lacking the coding sequences for the catalytic SET domain) combined with a *Jmjd1a*-null (*Jmjd1a*^Δ/Δ^) background. Embryonic gonads/mesonephroi at E11.5 were stained with antibodies against H3K9me2 and Nr5a1. The H3K9 methylation levels of Nr5a1-positive gonadal somatic cells were compared by FACS analysis (Fig 2A). Jmjd1a deficiency resulted in an increased H3K9me2 level in Nr5a1-positive gonadal somatic cells, indicating the substantial contribution of Jmjd1a to H3K9 demethylation (Fig 2B and 2C). Notably, introduction of the *GLP* mutation into the *Jmjd1a*^Δ/Δ^ background significantly reduced the H3K9me2 level in gonadal somatic cells (Fig 2B and 2C). Thus, we concluded that the GLP/G9a complex counteracts Jmjd1a-mediated H3K9 demethylation in the developing gonads in the sex-determining period.

### GLP/G9a complex catalyzes H3K9 dimethylation at the *Sry* locus

**Fig 3:**
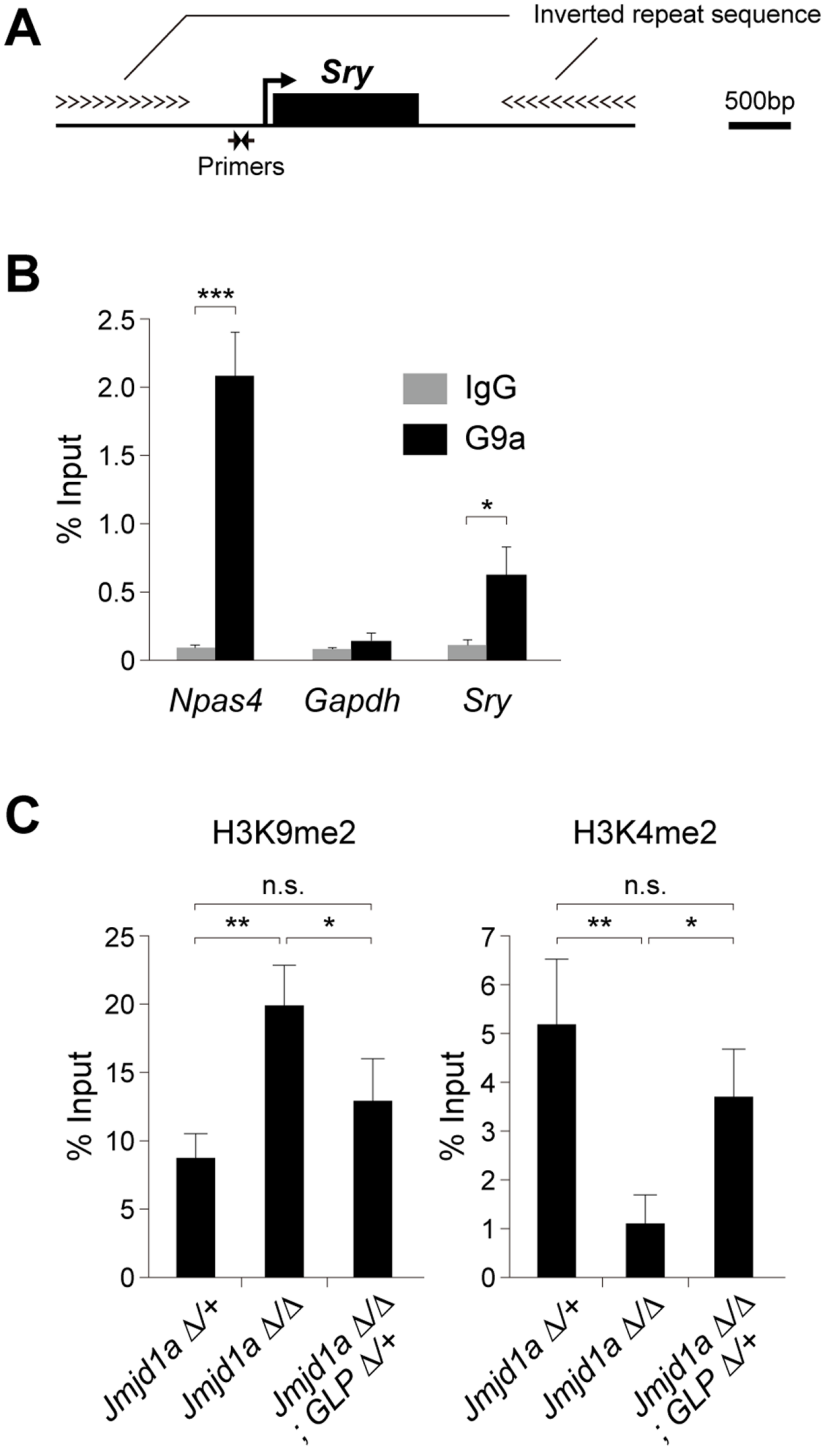
GLP/G9a complex catalyzes H3K9 dimethylation at the *Sry* locus. (A) Diagram of the *Sry* locus and primer location of the linear promoter region of *Sry*. (B) ChIP-qPCR analysis for G9a at the linear promoter region of *Sry*. Gonadal somatic cells were purified from E11.5 XY *Nr5a1*-*hCD271*-tg embryos, pooled, cross-linked, and then introduced into ChIP-qPCR analysis. We used *Npas4*, that had been identified as one of the target loci of G9a, as a positive control locus [16]. Data are presented as mean ± SD. * *P* < 0.05; *** *P* < 0.001. (C) ChIP-qPCR analyses for H3K9me2 (left) and H3K4me2 (right) at the *Sry* locus. Gonadal somatic cells of the indicated genotypes were purified according to the method described in S3 Fig, pooled for each genotype (2 to 4 embryos) and then subjected to native ChIP analysis. ChIP experiment was performed independently twice and gave similar results. Data are presented as mean ± SD. * *P* < 0.05; ** *P* < 0.01; n.s., not significant.

To examine the possible counteracting role of the GLP/G9a complex on Jmjd1a-mediated H3K9 demethylation at single gene level, especially at the *Sry* locus, chromatin immunoprecipitation (ChIP) analyses were performed. We previously demonstrated that Jmjd1a is enriched at the linear promoter region of *Sry* in E11.5 gonadal somatic cells [2] (Fig 3A). We purified gonadal somatic cells from E11.5 XY *Nr5a1*-*hCD271*-tg embryos and then subjected these cells to ChIP-qPCR analyses. As shown in Fig 3B, we found that G9a is also accumulated in the linear promoter region of *Sry*. We used *Npas4* as a positive control locus, that had been identified as one of the target loci of G9a [16]. We therefore concluded that H3K9 methyltransferase GLP/G9a complex and H3K9 demethylase Jmjd1a both target the *Sry* locus in embryonic gonadal somatic cells in the sex-determining period.

We next aimed to elucidate the impact of the *GLP* mutation on the H3K9me2 level of the *Sry* locus. Gonadal somatic cells were immunomagnetically purified from XY *Jmjd1a*^Δ/Δ^, *GLP*^Δ/+^, and *Nr5a1*-*CD271*-tg embryos for ChIP analysis (the experimental scheme is shown in S3 Fig). Importantly, the numbers of purified cells were similar among XY *Jmjd1a*^Δ/+^-, XY *Jmjd1a*^Δ/Δ^-, and XY *Jmjd1a*^Δ/Δ^;*GLP*^Δ/+^ gonads, indicating that these mutations did not affect gonadal somatic cell numbers (S3 Fig). Purified gonadal somatic cells were then subjected to native ChIP-qPCR analyses. Consistent with our previous study, Jmjd1a deficiency resulted in an increase of H3K9me2 at the *Sry* locus in E11.5 gonadal somatic cells, compared with the level in control cells (Fig 3C). Importantly, the increased level of H3K9me2 at the *Sry* locus was significantly rescued by the *GLP* mutation (Fig 3C). We also demonstrated the inverse relationship between H3K9me2 and H3K4me2 at the *Sry* locus. The latter is an epigenetic mark for transcriptionally activated chromatin (Fig 3C). Taking these findings together, the GLP/G9a complex is the bona fide enzyme responsible for H3K9 methylation at the *Sry* locus in E11.5 gonadal somatic cells.

### Jmjd1a- and GLP/G9a complex-mediated expression tuning is confined to *Sry* within the Y chromosome genes

**Fig 4:**
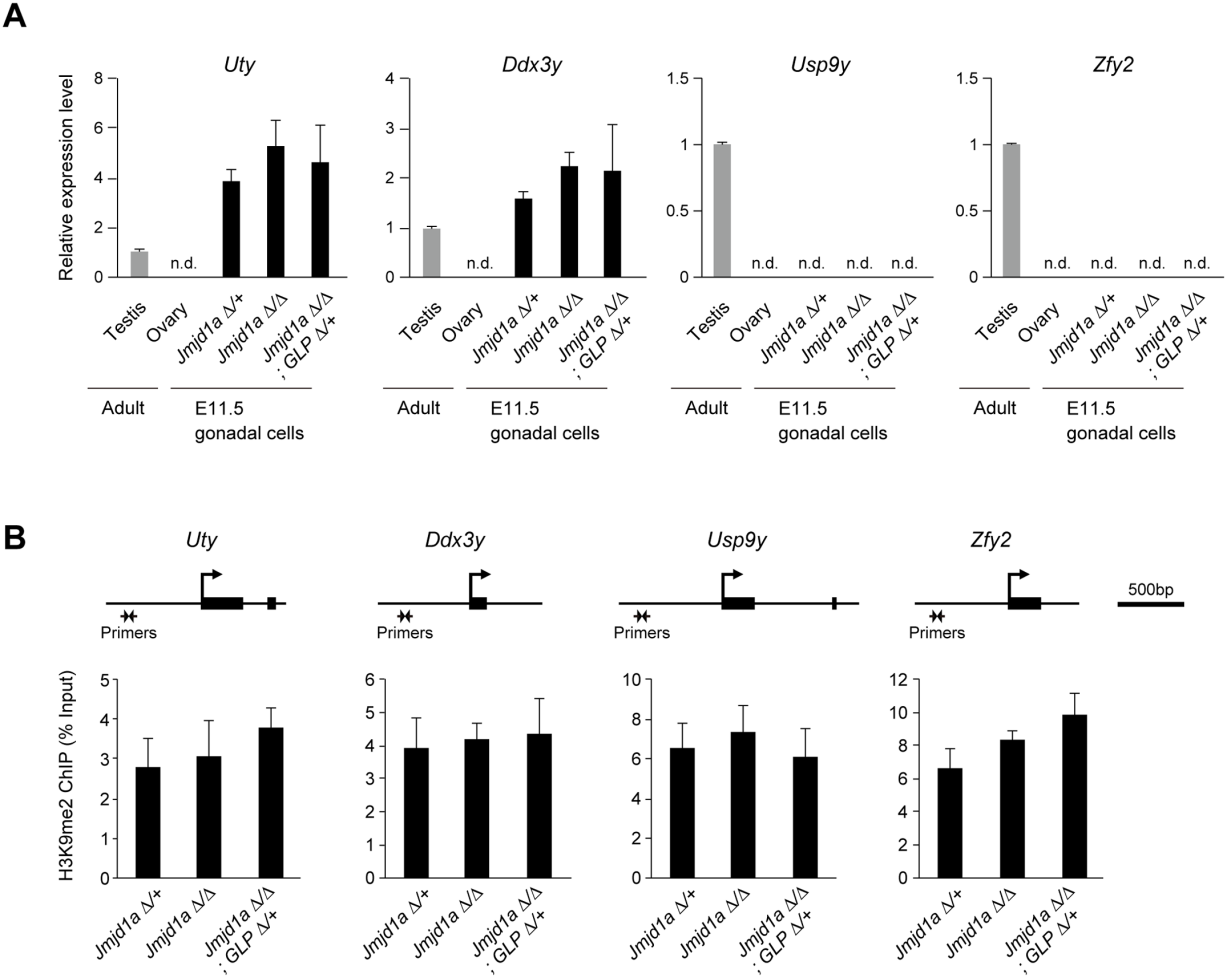
Jmjd1a- and GLP/G9a complex-mediated expression tuning is not extended to other genes on the Y chromosome. (A) E11.5 XY gonads of the indicated genotypes were dissected and subjected to mRNA expression analysis. We examined the expression levels of *Sry*-neighboring genes (*Uty*, *Ddx3y*, *Usp9y*, and *Zfy2*), which are located on the short arm of the Y chromosome. There was no significant difference in the expression levels of these genes between control and mutant gonads. mRNA expression levels in the adult testis (3 months) were defined as 1. All data are presented as mean ± SD. (B) ChIP-qPCR analyses for H3K9me2 at the *Uty*, *Ddx3y*, *Usp9y*, and *Zfy2* loci. Primer locations are shown at the top. There was no significant difference of the H3K9me2 levels between control and mutant gonads. All data are presented as mean ± SD.

We next elucidated whether the *Jmjd1a* mutation and/or *Jmjd1a*/*GLP* compound mutations may also induce transcriptional perturbation of Y chromosome genes other than *Sry*. Gonadal somatic cells were immunomagnetically purified from E11.5 embryos and then subjected to mRNA expression analysis. As shown in Fig 4A, we could not detect significant differences in the mRNA expression levels of *Sry*-neighboring genes, *Uty*, *Ddx3y*, *Usp9y*, and *Zfy2*, between control and mutant gonads. Accordingly, our previous microarray analysis showed that the expression levels of Y chromosome genes other than *Sry* were not affected by Jmjd1a deficiency [2]. Next, we evaluated the H3K9me2 levels of *Uty*, *Ddx3y*, *Usp9y*, and *Zfy2* by ChIP-qPCR analysis using purified E11.5 gonadal somatic cells (Fig 4B). Again, we could not detect significant differences in the H3K9me2 levels of these genes between control and mutant gonadal somatic cells. Taking these results together, Jmjd1a- and GLP/G9a complex-mediated expression tuning is highly confined to the *Sry* locus and is not extended to other genes on Y chromosome.

It is known that H3K4me3 and H3K9ac are enriched at the linear promoter region of *Sry* in E11.5 gonadal somatic cells [13]. To address whether *Jmjd1a* and/or *Jmjd1a/GLP* compound mutations may influence these modifications, we performed ChIP-qPCR analysis using purified E11.5 gonadal somatic cells. As shown in S4 Fig, neither *Jmjd1a* nor *Jmjd1a*/*GLP* compound mutations induced significant alterations of H3K4me3 and H3K9ac at the *Sry* locus. It is also known that CpG sequences of the linear promoter region of *Sry* are hypomethylated in embryonic gonads at the time of *Sry* expression [17]. To address whether Jmjd1a deficiency may influence DNA methylation of *Sry* promoter, we fractionated E11.5 gonadal somatic cells carrying the *Nr5a1-hCD271* transgene into hCD271-high and -low populations by FACS and introduced them into bisulfite sequence analysis. In control gonads, the DNA methylation level of *Sry* promoter was significantly low in the hCD271-high population compared to that of the hCD271-low population in E11.5 embryonic gonads (S4 Fig). These results are in accordance with a previous study [13]. We next compared DNA methylation levels of the *Sry* promoter of the hCD271-high population between *Jmjd1a*^Δ/+^ and *Jmjd1a*^Δ/Δ^ littermates. However, we could not find significant difference levels (S4 Fig). We therefore concluded that *Jmjd1a* mutation did not induce a significant alteration of DNA methylation at the *Sry* locus.

### The *GLP* mutation rescues the reduced expression of *Sry* in Jmjd1a-deficient embryos

**Fig 5:**
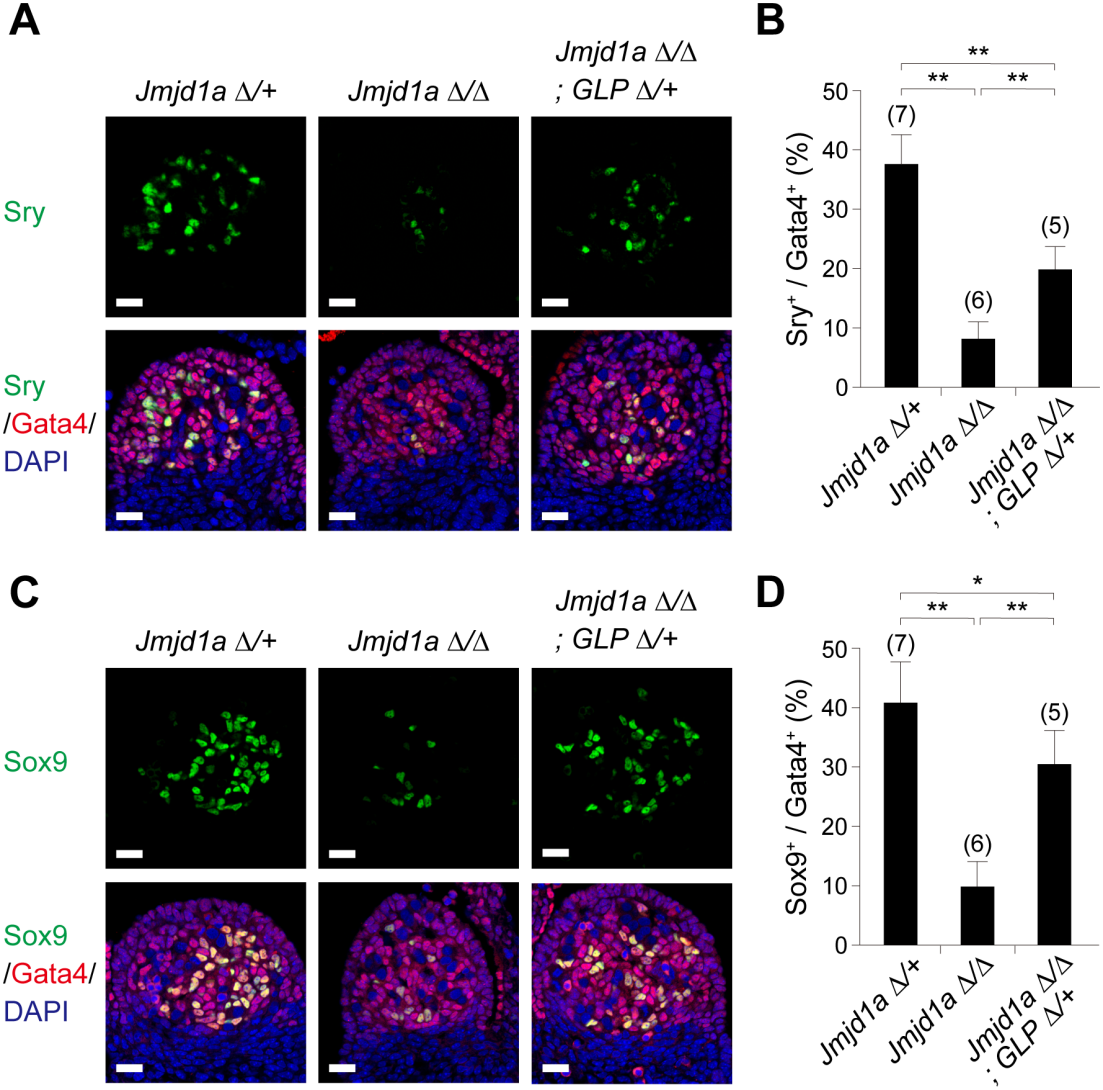
The *GLP* mutation rescues the reduced expression of Sry in Jmjd1a-deficient embryos. (A, C) Co-immunostaining profiles of Sry (A) and Sox9 (C) with a gonadal somatic cell marker, Gata4, in the center regions of E11.5 gonads of the indicated genotypes. Scale bar, 20 μm. (B, D) The ratios of cells positive for Sry (B) and Sox9 (D) to Gata4-positive cells in E11.5 gonads of the indicated genotypes. Numbers of examined animals are shown above the bars. All data are presented as mean ± SD. * *P* < 0.05; ** *P* < 0.01.

*Sry* activation is the first event in mammalian sex differentiation. In mice, sufficient and temporally accurate expression of *Sry* in sexually undifferentiated gonads at E11.5 is critical for triggering the testis-determining pathway [18]. To address whether the *GLP* mutation also rescues the perturbed expression of *Sry* in Jmjd1a-deficient gonads, we examined Sry expression by co-immunofluorescence analysis for Sry and Gata4, a marker of gonadal somatic cells. As shown in Fig 5A and 5B, the number of Sry-positive cells was reduced to approximately 25% in XY *Jmjd1a*^Δ/Δ^ gonads at E11.5. The number of Sry-positive cells was significantly, although not completely, rescued in XY *Jmjd1a*^Δ/Δ^;*GLP*^Δ/+^ littermates, indicating that the GLP/G9a complex and Jmjd1a antagonistically tune Sry expression in E11.5 gonadal somatic cells. We also performed expression analysis of Sox9, a downstream target of Sry, in E11.5 gonads (Fig 5C and 5D). The number of Sox9-positive cells was also increased by the *GLP* mutation. Interestingly, the *GLP* mutation had a more profound effect on the increase of Sox9-positive cells compared with that of Sry-positive cells, presumably reflecting the activation of a non-cell-autonomous pathway of Sox9 expression [19].

### The *GLP* mutation rescues abnormal sex differentiation of XY Jmjd1a-deficient embryos

**Fig 6:**
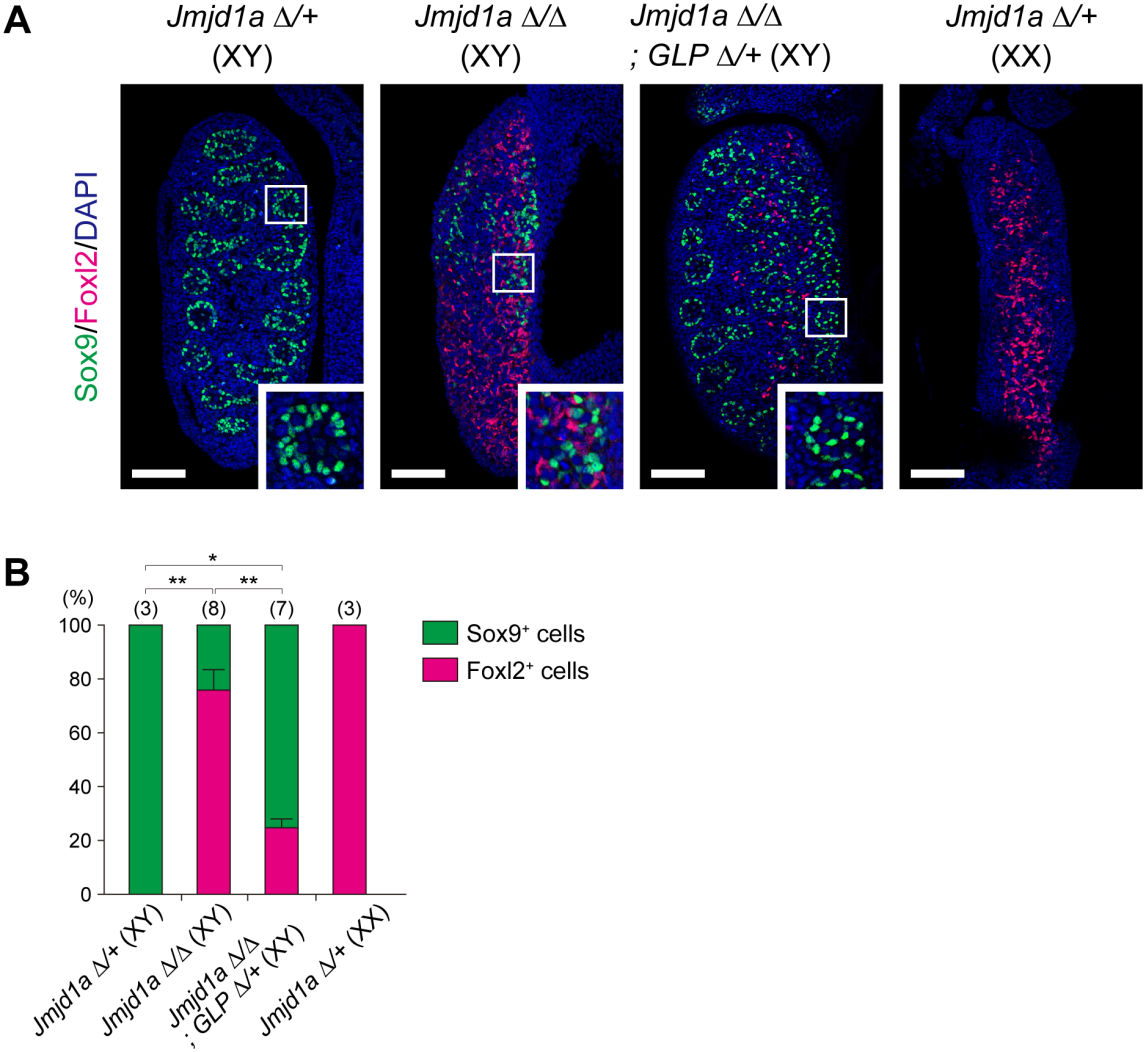
The *GLP* mutation rescues abnormal sex differentiation of XY Jmjd1a-deficient embryos. (A) Evaluation of sex differentiation of E13.5 embryonic gonads by immunofluorescence analysis. Sox9 and Foxl2 mark testicular Sertoli and ovarian somatic cells, respectively. The enlarged box indicates that the *GLP* mutation rescued the tubule-like structure that was absent in XY *Jmjd1a*^Δ/Δ^ gonads. Scale bar, 200 μm. (B) Quantification of Sox9- and Foxl2-positive cells in E13.5 gonads of the indicated genitypes. Numbers of embryos examined are shown above the bars. Data are presented as mean ± SD. * *P* < 0.05; ** *P* < 0.01.

To examine embryonic gonadal cell differentiation just after sex determination, we investigated the expression of the testicular Sertoli cell marker Sox9 and the ovarian somatic cell marker Foxl2 in XY *Jmjd1a*^Δ/Δ-^ and XY *Jmjd1a*^Δ/Δ^;*GLP*^Δ/+^ embryonic gonads at E13.5 (Fig 6A). Control XY gonads had Sox9-positive cells and did not contain Foxl2-positive cells. Furthermore, multiple tubule-like structures were found in control XY gonads. On the other hand, Jmjd1a-deficient XY gonads were ovotestes containing not only Sox9- but also Foxl2-positive cells and had no tubule-like structures. As shown in Fig 6A and 6B, the *GLP* mutation restored the number of Sox9-positive cells and testicular tubule formation, both of which were perturbed by Jmjd1a deficiency, indicating that the *GLP* mutation successfully rescued gonadal sex differentiation of Jmjd1a-deficient embryos.

GLP and G9a form a heterodimer, which is essential for H3K9 methylation *in vivo*. We thus also performed epistatic analyses between *G9a* and *Jmjd1a* in mouse sex development. Notably, a *G9a* heterozygous mutation did not rescue the sex-reversal phenotype of XY Jmjd1a-deficient embryos (S5 Fig). Our previous studies indicated that GLP, but not G9a, is a limiting factor controlling the amount of GLP/G9a holoenzyme complex [10]. Consistent with this, we found that the *GLP* heterozygous mutation induced a significant reduction of G9a protein in embryonic gonads (S6 Fig). Thus, the decreased level of H3K9me2 associated with the *GLP* heterozygous mutation may be attributable to the reduction in the amount of the GLP/G9a complex.

We had previously established *GLP*-tg mice [20]. In this line, cDNA for Flag-tagged GLP was inserted in the *Rosa26* locus and was designed to be expressed ubiquitously depending on an artificial CAG promoter [20]. To learn whether the overexpression of *GLP* affects sex determination, we compared the expression levels of *Nr5a1*, *Sry*, and *Sox9* in gonads of *GLP*-tg XY embryos at E11.5. As shown in S7 Fig, although the amount of *GLP* transcript was actually elevated in XY *GLP*-tg gonads of E11.5 embryos, those of *Nr5a1*, *Sry*, and *Sox9* transcripts were indistinguishable between XY control and XY *GLP*-tg gonads (S7 Fig). A possible explanation is that unidentified limiting factor(s) other than GLP may be required for the GLP/G9a complex to exert its function in the developing gonads.

### The *GLP* mutation rescues XY sex reversal in Jmjd1a-deficient adult mice

**Fig 7:**
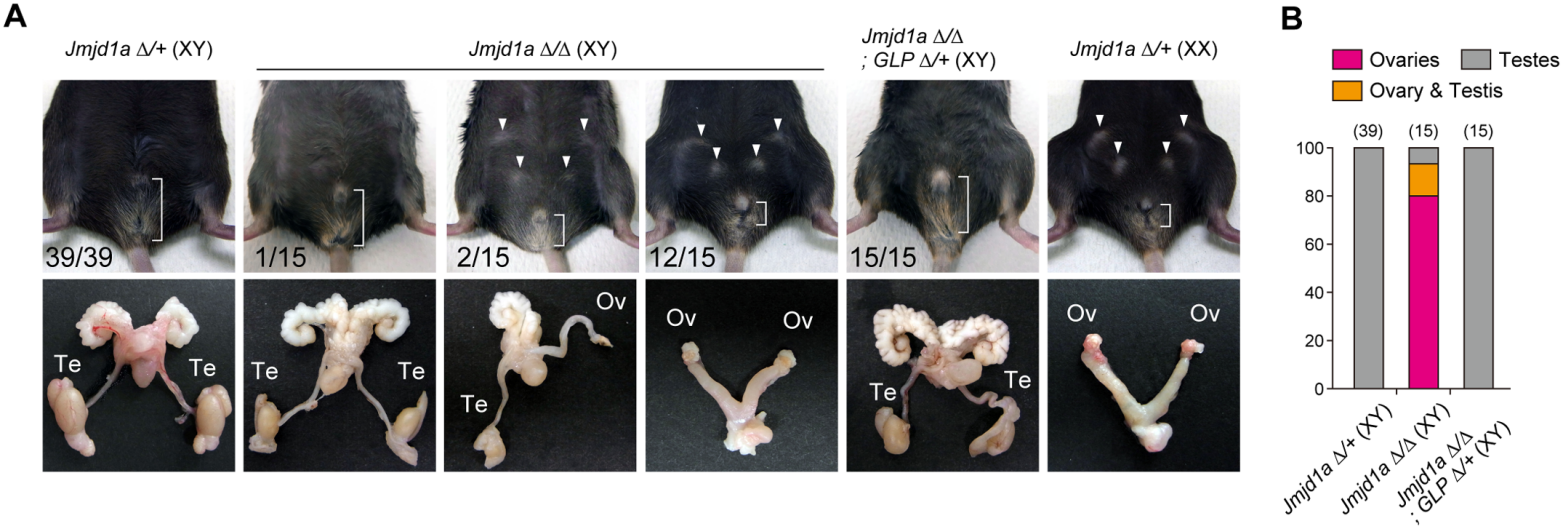
The *GLP* mutation rescues XY sex reversal in Jmjd1a-deficient adult mice. (A) External genitalia (upper) and gonads and genital tracts (lower) of 3-months-old mice of the indicated genotypes. Arrowheads represent mammary glands. The distance between anus and penis or vagina is indicated. Frequencies are presented in the lower left corner. Te, testis; Ov, ovary. (B) Frequency analysis of abnormal sex differentiation of 3-months-old mice, determined by examining internal genitalia. Numbers of animals examined are shown above the bars.

We finally verified the impact of the *GLP* mutation on the sex-reversal phenotype of Jmjd1a-deficient mice in adults. Consistent with our previous study [2], XY mice lacking *Jmjd1a* alone were frequently sex-reversed; for example, analysis of the external genitalia revealed that of 15 XY *Jmjd1a*^Δ/Δ^ animals, one carried male-type genitalia, two carried intersex-type genitalia with a micropenis and well-developed mammary glands, and another carried typical female-type genitalia (Fig 7A). In addition, analysis of the internal genitalia demonstrated that one exhibited bilateral testes, two exhibited a testis and an ovary, and another had two ovaries. In contrast, all XY *Jmjd1a*^Δ/Δ^;*GLP*^Δ/+^ littermates exhibited male-type external genitalia with bilateral testes (Fig 7B). Taking these findings together, we conclude that the *GLP* mutation completely rescued the adult sex-reversal phenotype of Jmjd1a-deficient XY mice.

### Embryonic administration of the GLP/G9a inhibitor UNC0642 reverses aberrant sex development of Jmjd1a-deficient mice

**Fig 8:**
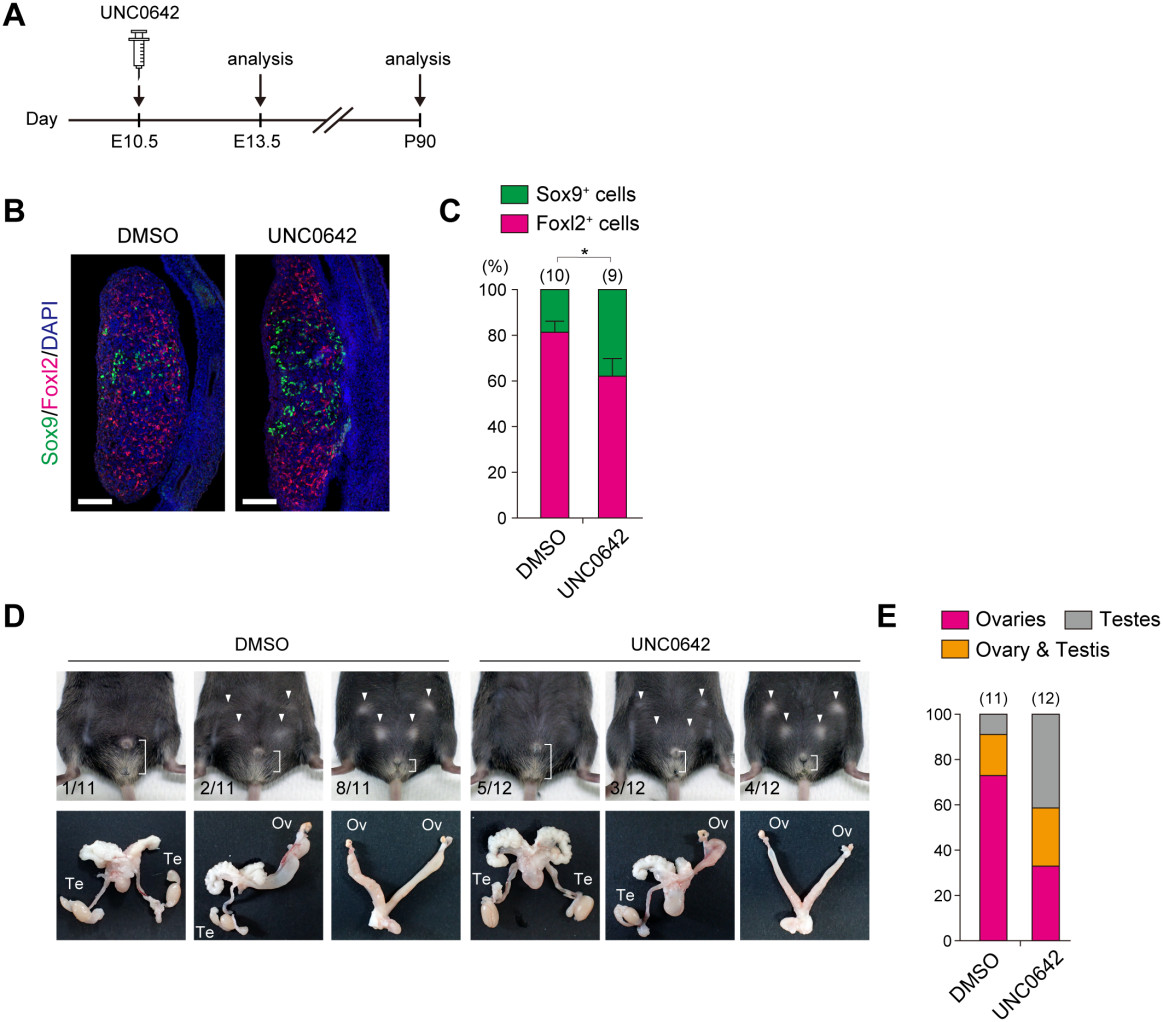
Embryonic administration of the GLP/G9a inhibitor UNC0642 rescues aberrant sex development of Jmjd1a-deficient mice. (A) Experimental scheme of UNC0642 treatment. 0.5 mg of UNC0642 was intraperitoneally injected into pregnant females carrying E10.5 Jmjd1a-deficient embryos, and the subsequent gonadal differentiation of E13.5 embryos (B) and 3-months-old adults (D) was examined. (B) Immunofluorescence analysis of sex differentiation of E13.5 embryonic gonads using antibodies against Sox9 and Foxl2. Scale bar, 200 μm. (C) Quantification of Sox9- and Foxl2-positive cells in E13.5 gonads. Numbers of embryos examined are shown above the bars. Data are presented as mean ± SD. * *P* < 0.05. (D) External genitalia (upper) and gonads and genital tracts (lower) of UNC0642-treated (right) and solvent-treated (left) XY Jmjd1a-deficient animals. Arrowheads represent mammary glands. The distance between anus and penis or vagina is indicated. Frequencies are presented in the lower left corner. Te, testis; Ov, ovary. (E) Frequency analysis of abnormal sex differentiation of 3-months-old mice, determined by examining the internal genitalia. Numbers of animals examined are shown above the bars.

Our genetic experiments revealed that modulation of H3K9 methyltransferase activity of the GLP/G9a complex might be therapeutically effective for rescuing the aberrant sex development of Jmjd1a-deficient mice. We next aimed to rescue the phenotype in a different way using UNC0642, a chemical inhibitor of the GLP/G9a complex, which was recently developed by Liu et al. [21]. The experimental scheme of UNC0642 administration to Jmjd1a-deficient embryos is shown in Fig 8A. Briefly, 0.5 mg of UNC0642 was intraperitoneally injected once into pregnant females carrying E10.5 Jmjd1a-deficient embryos, and then the gonadal differentiation of the embryos was examined. As shown in Fig 8B and 8C, UNC0642 administration to Jmjd1a-deficient embryos resulted in a significant increase in the number of Sox9-positive male somatic cells at E13.5, while solvent only did not (compare with Fig 6B). This indicates that UNC0642 partially, but significantly, rescued the gonadal sex differentiation of Jmjd1a-deficient embryos after sex determination. We next investigated the impact of the embryonic administration of UNC0642 on the sex development of Jmjd1a-deficient mice by examining the external and internal genitalia of adult mice (Fig 8D). Although partially or completely sex-reversed mice were still found in the UNC0642-administered Jmjd1a-deficient mice, five out of 12 UNC0642-administered animals carried bilateral testes. In contrast, only one out of 11 animals in the solvent-injected control group exhibited bilateral testes (Fig 8D and 8E). Taking these findings together, we conclude that administration of UNC0642 into E10.5 embryos successfully rescued the subsequent sex development of Jmjd1a-deficient mice.

## Discussion

Here, we identified GLP/G9a H3K9 methyltransferase complex as an enzyme counteracting Jmjd1a-mediated H3K9 demethylation at the *Sry* locus in gonadal somatic cells. To our knowledge, this is the first study to identify the set of histone methyltransferase and demethylase that in combination account for stage- and cell-type-specific gene regulation in mammalian development.

**Fig 9:**
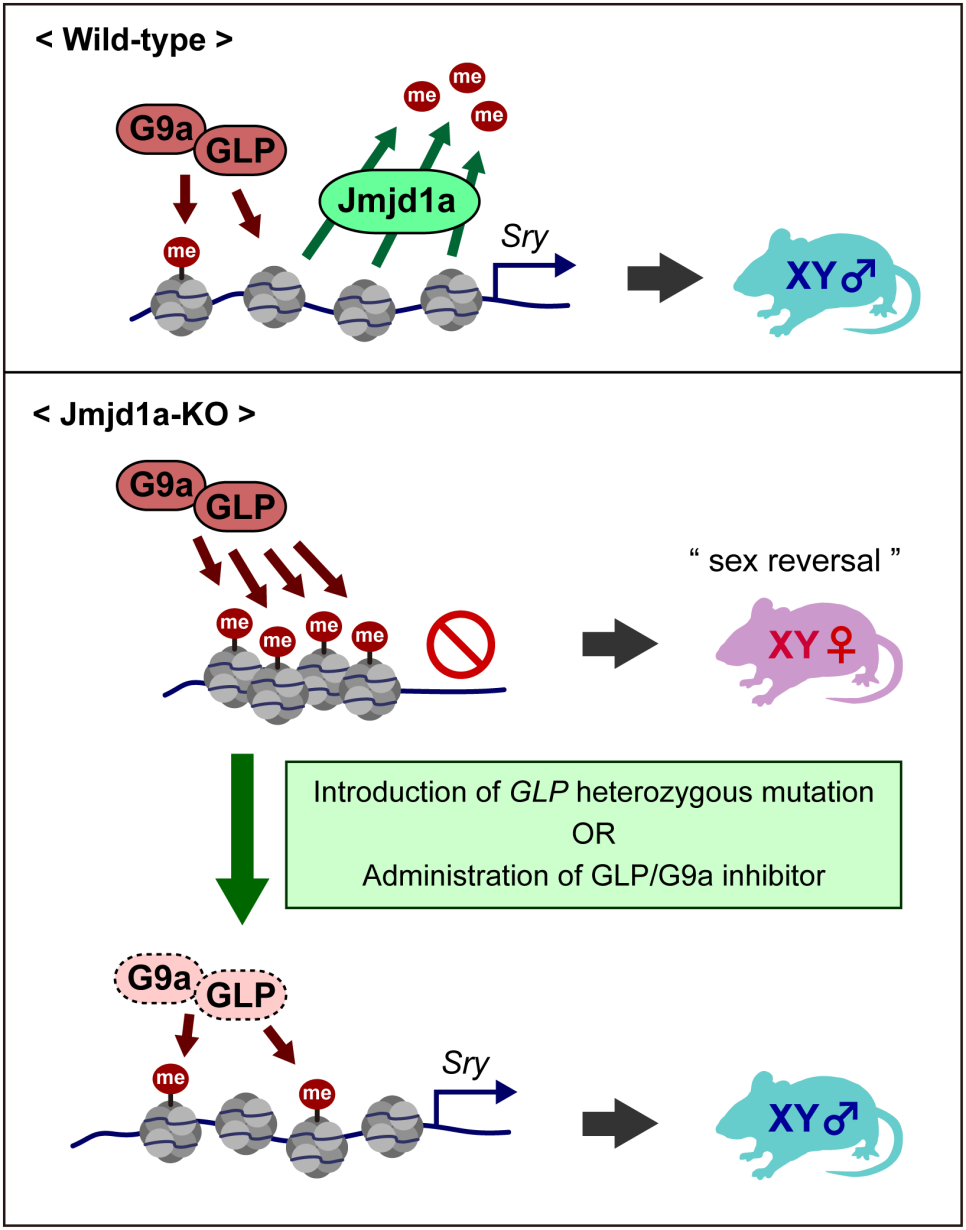
Fine-tuning of *Sry* expression is achieved by the balance in activities between H3K9 demethylase Jmjd1a and H3K9 methyltransferase GLP/G9a complex. In wild-type embryonic gonads, Jmjd1a removes H3K9 methylation marks, which were deposited by GLP/G9a complex, from the *Sry* locus, thereby ensuring *Sry* activation. In Jmjd1a-deficient embryonic gonads, the absence of Jmjd1a results in the GLP/G9a complex-mediated H3K9 hypermethylation at the *Sry* locus, thereby compromising *Sry* expression and causing male-to-female sex reversal. Normalization of the H3K9 methylation balance of the *Sry* locus by a genetic or a pharmacological approach rescues the aberrant sex development of Jmjd1a-deficient mice by restoring *Sry* expression.

Our data show that the molecular balance of the GLP/G9a complex and Jmjd1a is a critical factor for the tuning of *Sry* expression (Fig 9). We previously showed that G9a and GLP are expressed in almost all adult tissues in mice [9, 10]. On the other hand, previous studies demonstrated that Jmjd1a is expressed in a tissue- and developmental stage-specific manner [22–24]. Considering that the expression of *Sry* is suppressed in almost all adult tissues in mice, this *Sry* silencing might be explained, at least in part, by the robust H3K9 methylation of the GLP/G9a complex and the absence of H3K9 demethylase in these tissues. We previously demonstrated the temporally specific expression of *Jmjd1a* in embryonic gonadal somatic cells, which reaches a plateau around E11.5 [2]. In addition, we demonstrated the cell-type-specific expression of *Jmjd1a* in this study, as *Jmjd1a* transcript was substantially enriched in the Nr5a1-high population of E11.5 embryonic gonads (Fig 1C). High levels of *Jmjd1a* expression may overcome GLP/G9a complex-mediated H3K9 methylation, thereby inducing *Sry* expression in the pre-Sertoli cells.

Although the *GLP* mutation significantly rescued the perturbed *Sry* expression in Jmjd1a-deficient embryonic gonads, the *Sry*-positive cell population in *Jmjd1a*^Δ/Δ^;*GLP*^Δ/+^ gonads remained approximately half of that detected in control gonads (Fig 5B). Therefore, it is a surprising finding that the *GLP* mutation completely rescued the sex reversal of Jmjd1a-deficient XY mice in adults (Fig 7). The *GLP* mutation reduces global H3K9me2 levels of Jmjd1a-deficient gonadal somatic cells (Fig 2). Thus, it is also possible that GLP/G9a complex has a role in the sex differentiation pathway, independent of *Sry* regulation. In this regard, the *GLP* mutation may inhibit the ovarian development pathway in Jmjd1a-deficient gonads. Although we could not rule out this possibility, it seems likely that the restored *Sry* expression is a primary cause for the rescue of the sex reversal of Jmjd1a-deficient XY mice. Because there is a certain threshold level for *Sry* expression to induce the male pathway [18], it is conceivable that *Sry* expression substantially exceeds the threshold level as a result of the *GLP* mutation in the Jmjd1a-deficient background, thereby conferring profound effects on the subsequent male pathway.

Eset is known as an enzyme responsible for tri-methylation of H3K9 [6]. A previous study demonstrated that Eset is expressed in embryonic gonads [25]. Accordingly, we also confirmed the expression of *Eset* in E11.5 XY gonadal somatic cells by RT-qPCR analysis [2]. We introduced the *Eset* heterozygous mutation into the *Jmjd1a*-mutant background, and then the sex development of XY *Jmjd1a*^Δ/Δ^; *Eset*^Δ/+^ embryos was examined (S8 Fig). Consequently, we could not find any profound effect of the *Eset* mutation on the perturbed sex development of Jmjd1a-deficient mice (S8 Fig). Our previous study indicated that the H3K9me3 level of *Sry* was unchanged by Jmjd1a deficiency in E11.5 embryonic gonads. Altogether, it seems likely that Jmjd1a-mediated H3K9 demethylation does not counteract Eset-mediated H3K9 tri-methylation, at least in the *Sry* locus.

Previous studies revealed the enrichment of H3K4me3/H3K9ac and that low levels of CpG methylation are characteristic in the *Sry* promoter region of gonadal somatic cells during the sex-determining period [17] [13]. In this study, we demonstrated that Jmjd1a deficiency and/or the increased level of H3K9me2 did not affect H3K4me3/H3K9ac and CpG methylation levels of the *Sry* locus (S4 Fig). On the other hand, it seems likely that H3K9me2 and H3K4me2 marks are exclusively deposited mutually and these marks exert antagonistic functions for transcription, at least in the *Sry* locus. Jmjd1a deficiency and/or the increased levels of H3K9me2 resulted in the decrease of H3K4me2 of this locus, concomitantly with the suppression of *Sry* [2], also shown in Fig 3. The fact that Jmjd1a deficiency result in the loss of H3K4me2, but not H3K4me3, in the *Sry* locus warrants further discussion. It is one possible explanation that Jmjd1a or Jmjd1a-mediated H3K9 hypomethylation may prevent the accession of specific enzyme(s) responsible for H3K4me2 demethylation.

We have demonstrated that just a single administration of GLP/G9a inhibitor to E10.5 embryos significantly rescues the aberrant sex development of Jmjd1a-deficient mice. Mutation, silencing, or downregulation of histone methylation “erasers” was found in several types of cancer [26]. Our experiments suggest a new therapeutic strategy, in which diseases arising from the dysfunction of an epigenetic “eraser” can be rescued by blocking the activity of the counteracting epigenetic “writer.”

## Materials and methods

### Ethics statement

Animal experiments were performed under the animal ethical guidelines of Tokushima University and Kyoto University. The Ethics Committee of Tokushima University for Animal Research (Approval number: 14108) and the Animal Experimentation Committee of Kyoto University (Approval number: A12-6-2) approved this study.

### Animals

Mouse lines of *GLP*^Δ/+^, *G9a*^Δ/+^, *Jmjd1a*^Δ/+^, and *Nr5a1-hCD271*-tg were sequentially backcrossed to C57BL/6J, and then the F_5_ or later generation was used. Since the sex reversal frequencies of Jmjd1a-deficient mice were dependent on the origin of the Y chromosome [2], we used mice carrying a Y chromosome of CBA origin in this study. However, we only used mice carrying a Y chromosome of B6 origin in the experiments shown in S5 Fig.

### Antibodies

Guinea-pig polyclonal antibodies against Sry were generated by the immunization of bacterially expressed 6xHis-tagged Sry (residues 82–395, NP_035694). Additional antibodies used in this study were as follows: goat anti-Gata4 (Santa Cruz, C-20), rabbit anti-Sox9 (Millipore, AB5535), goat anti-Foxl2 (Abcam, ab-5096), mouse anti-LNGFR (Miltenyi Biotec), rabbit anti-Jmjd1a [2], mouse anti-G9a (Perseus Proteomics, 8620A), mouse anti-GLP (Perseus Proteomics, B0422), rabbit anti-G9a (CST, #3306), mouse anti-H3K9me2 [27], mouse anti-H3K4me2 [27], mouse anti-H3K4me3 [27], mouse anti-H3K9ac [27], and rabbit anti-Nr5a1 (a gift from Dr. K. Morohashi).

### Histology and immunohistochemistry

Tissues were fixed in either Bouin’s solution or 4% paraformaldehyde, embedded in paraffin, and cut into 4-μm sections. For histological analysis, sections were stained with hematoxylin/eosin or hematoxylin/PAS. For immunohistochemistry, sections were deparaffinized and rehydrated, and then autocleaved at 105°C for 5 min in 10 mM citric acid buffer (pH 6.0). To quench endogenous peroxidase, the sections were treated with 0.3% (v/v) hydrogen peroxide. After blocking with TBS containing 2% skim milk and 0.1% Triton-X100 at room temperature for 1 h, sections were incubated with primary antibodies overnight at 4°C. For fluorescence staining, the sections were incubated with Alexa-conjugated secondary antibodies (Life Technologies) at room temperature for 1 h and counterstained with DAPI. The sections were mounted in Vectashield (Vector) and observed with a confocal laser scanning microscope (LSM700, Carl Zeiss).

### Flow cytometry and cell sorting

Isolated gonads and mesonephroi from E11.5 embryos were digested with Accutase (Nacalai) to obtain a single cell suspension. For flow cytometric analysis, cells were fixed with 2% paraformaldehyde (PFA) in PBS for 10 min, permeabilized with ice-cold ethanol for 20 min, and blocked with 0.5% skim milk in PBS for 1 h. They were then stained with primary antibodies overnight at 4°C and subsequently incubated with Alexa-conjugated secondary antibodies (Life Technologies) for 1 h at room temperature. Data were collected using FACSCanto 2 (BD Bioscience) and analyzed with FlowJo software (TreeStar). For FACS sorting, cells were stained with FITC-labeled anti-LNGFR and sorted based on fluorescence intensity using FACS Aria 2 (BD Bioscience) as shown in S2 Fig.

### ChIP analysis

The experimental scheme for ChIP analysis is shown in S3 Fig. Briefly, two-cell embryos were prepared by *in vitro* fertilization using sperm derived from *Jmjd1a*^Δ/+^;*GLP*^Δ/+^;*Nr5a1-hCD271*-tg males and eggs derived from *Jmjd1a*^Δ/+^ females, and then transferred to pseudopregnant recipients. Gonadal somatic cells were purified from embryos that had developed to tail somite stage 17–19, as described previously [2, 14]. For native ChIP analysis of histone modifications, purified cells were pooled (*n* = 2–4 per genotype) and subjected to ChIP analysis following a protocol described previously [15], with minor modifications. Briefly, cells were suspended in 5 μl of 0.3 M sucrose-containing buffer 1 (60 mM KCl, 15 mM NaCl, 5 mM MgCl_2_, 0.1 mM EGTA, 0.5 mM dithiothreitol, 0.1 mM PMSF, 3.6 ng/ml aprotinin, 15 mM Tris–HCl, pH 7.5) and lysed by the addition of 5 μl of 0.3 M sucrose-containing buffer 1 with 0.8% NP40 on ice for 10 min. After the addition of 80 μl of 1.2 M sucrose-containing buffer 1, the chromatin fraction was collected as pellets by centrifugation. These pellets were digested with micrococcal nuclease (MNase) (0.02–0.05 U, Takara) in 10 μl of MNase digestion buffer (0.32 M sucrose, 4 mM MgCl_2_, 1 mM CaCl_2_, 0.1 mM PMSF, 50 mM Tris–HCl, pH 7.5), using Thermo Mixer (Eppendorf) at 37°C and 1000 rpm for 15 min, and then digestion was stopped with EDTA. Supernatant was obtained by centrifugation and incubated with anti-H3K9me2-, anti-H3K9ac-, anti-H3K4me2-, or anti-H3K4me3-coated magnetic beads (Dynabeads Protein G, Invitrogen) in 50 μl of incubation buffer (50 mM NaCl, 5 mM EDTA, 0.1% NP40, 0.1 mM PMSF, 20 mM Tris–HCl, pH 7.5), at 4°C for 2 h. DNA was extracted from the immune complexes according to the standard protocol and then analyzed by real-time PCR using primers specific for Y chromosome genes (*Sry*, *Uty*, *Ddx3y*, *Usp9y*, and *Zfy2*). For cross-link ChIP analysis of G9a, purified gonadal somatic cells from 20 embryos (approximately 8 × 10^5^ cells) were pooled and combined with 5 × 10^6^ cells of female mouse embryonic fibroblasts, cross-linked with 25 mM DSG (Thermo Fisher Scientific) and 1% formaldehyde, and applied to ChIP analysis with rabbit anti-G9a antibody following a protocol described previously [2].

### Bisulfite sequencing analysis

Genomic DNA was isolated using the All DNA/RNA Micro kit (QIAGEN). Genomic DNA was treated with sodium bisulfite using the MethylEasy Xceed Rapid DNA Bisulfite Modification Kit (Human Genetic Signatures) following the manufacturer’s instructions. The bisulfite-treated DNA was PCR-amplified using the primer pair 5′-TTTATATTGGGTTATAGAGTTAGAATAGAT-3′ and 5′-CCAAAATATACTTATAACAAAAATTTTAAT-3′. PCR products were subcloned into the pGEM-T Easy vector (Promega) and sequenced.

### Primers

The primer sets used in ChIP-qPCR analysis were as follows: *Sry* linear prom.-f (5′-TGGTCAGTGGCTTTTAGCTCT-3′) and *Sry* linear prom.-r (5′-AGATGTGATGCAAAGAGAAACA-3′) for *Sry*, Npas4 ChIP F (5′-CTATGGCCATTTCAGCACCG-3′) and Npas4 ChIP R (5′-AGCTGTTCGACGTCCTGAAG-3′) for *Naps4*, Gapdh ChIP F (5′-TTGCTTAGGCCTTCCTTCTTC-3′) and Gapdh ChIP R (5′-CATCACCTGGCCTACAGGATA-3′) for *Gapdh*, ChIP-Uty-F (5′-CCTTTGTGAGGGACTGTTCA-3′) and ChIP-Uty-R (5′-CCACTCAACCACATCAAACC-3′) for *Uty*, ChIP-Ddx3y-F (5′-ACAATTCCACAACCCAAGGT-3′) and ChIP-Ddx3y-R (5′-AGGTTTCAGCCCACTCATTT-3′) for *Ddx3y*, ChIP-Usp9y-F (5′-AAGGGACACACAGTTCTCCA-3′) and ChIP-Usp9y-R (5′- CTTGTGAGAAGGGACTGAGG-3′) for *Usp9y*, ChIP-Zfy2-F (5′- AGGCAGTCTTAGATGCGAAA-3′) and ChIP-Zfy2-R (5′- TCCTGACTCACAACAACAGC-3′) for *Zfy2*. The primer sets used in RT-qPCR analysis were as follows: Gapdh RT-PCR F (5′-ATGAATACGGCTACAGCAACAGG-3′) and Gapdh RT-PCR R (5′-CTCTTGCTCAGTGTCCTTGCTG-3′) for *Gapdh*, Ad4BP-e2-F (5′-TTGTCGACTGGGCACGAAGGTGCAT-3′) and Ad4BP-e2-R (5′-GCAGCTCGCTCCAACAGTTCTGCAG-3′) for *Nr5a1*, Sry-5-SD (5′-TACCTACTTACTAACAGCTGACATCAC-3′) and Sry-3-SD (5′-TGTCATGAGACTGCCAACCACAGGG-3′) for *Sry*, TSGA-EX 21F (5′-ACTCCAGAGGATCGGAAATATGGGACC-3′) and TSGA-EX 21R (5′-GGGAATTCCCACATAAACCATGACATTGGC-3′) for *Jmjd1a*, GLP-RT-1B (5′-AACCCAACCTTGTGCCTGTGCGAG-3′) and GLP-RT-2 (5′-CGAGCTGCTCCCCAGCCTGAATCAG-3′) for *GLP*, G9a-RT-1B (5′-ACCCCAACATCATCCCTGTCCGGG-3′) and G9a-RT-2 (5′-GTCCCAGAATCGGTCACCGTAGTC-3′) for *G9a*, Sox9-RT-F (5′-AGGAAGCTGGCAGACCAGTA-3′) and Sox9-RT-R (5′-CGTTCTTCACCGACTTCCTC-3′) for *Sox9*, Uty-RT-F (5′-AAGGCGCTTTGTGGATTAGA-3′) and Uty-RT-R (5′-CTGATTCCACTTTTCCTTCAGC-3′) for *Uty*, Ddx3y-RT-F (5′- TTGGTCTTGACCTGAAATCATCA-3′) and Ddx3y-RT-R (5′- GCTTCCCTCTGGAATCACGA-3′) for *Ddx3y*, Usp9y-RT-F (5′- CTTGGTCCCAAATTGCAAGC-3′) and Usp9y-RT-R (5′- TCGGATGGCTTCTTGTCTTG-3′) for *Usp9y*, Zfy2-Rt-F (5′- GCTTAAGACCTCCAGCAAAAG-3′) and Zfy2-Rt-R (5′- CCGGTCTCTGGCTTTAATGT-3′) for *Zfy2*. The primer sets used for genotyping were as follows: GLP-6570F (5′-CTGTCCAGTTCCCGATTTTCAAGACTGC-3′) and GLP-5936R (5′-GTCCCACTGGCCACACTGGCAATTC-3′) for detection of the *GLP*^Δ^ allele; TSGA-G1475R (5′-GAACTGCACCATTAGCTGTCACTTCC-3′), TSGA-1980F (5′-CATGCAGTGAAAGATGCAGTTGCTA-3′), and TSGA-6410F-NheSac (5′-CTAAATATCAAGGCTAGCGAGCTCG-3′) for detection of the *Jmjd1a*^Δ^ allele; Sf1-1741F (5′-CACAGACCAGGGCAATCCCAAGCCA-3′) and pMACS-LI 2264R (5′-GTCGGAGAACGTCACGCTGTCCAG-3′) for *Nr5a1-hCD271*-tg; Rbmy1a1-F (5′-AATATGCCAAGAGGAGAGCCGGCGTCTTCC-3′) and Rbmy1a1-R2 (intron) (5′-CCAAGTTGTTGTGGCATTTGGACATC-3′) for detection of the Y chromosome; and GE28R (5′-GCTCCAGGGCGATGGCCTCCGCTGAATGC-3′), GI27-2F (5′-CGGGACAGGGTTTCTCTGTGTAGTCC-3′), and GI-25F (5′-CTGCACGCTGCCTAGATGGAGCATG-3′) for detection of the *G9a*^Δ^ allele.

### GLP/G9a inhibitor UNC0642

Pregnant females at E10.5 were administered 0.5 mg of UNC0642 (Tocris) dissolved in 30 μl of DMSO and mixed with 17.5 μl of ethanol, 52.5 μl of castor oil, and 100 μl of PBS.

### Generation of *Eset*-mutant mice using a CRISPR/Cas9 system

*Eset*-mutant mice were produced by electroporating *Cas9* mRNA and gRNA into mouse zygotes according to a protocol published recently [28]. Briefly, 400 ng/μl *Cas9* mRNA and 100 ng/μl of each gRNA targeting the genomic sequences of *Eset* (shown in S8 Fig) were introduced into zygotes (C57BL/6J × C57BL/6J) by electroporation using Genome Editor GEB15 (BEX, Tokyo, Japan). The electroporation conditions were four pulses of 30 V (3 ms ON + 97 ms OFF). The surviving two-cell-stage embryos were transferred to the oviducts of pseudopregnant females. Genotyping of the generated mice was performed using the primer pair 5′-CCCTGGCTGTCCTAGAACTCAC-3′ and 5′-AGGGTTCATTCAGGCTACAAAG-3′.

### Statistics

One-way analysis of variance (one-way ANOVA) and Tukey’s honestly significant difference test were used for statistical analysis.

## Supporting information

S1 Fig H3K9 methylation profiles of Jmjd1a-deficient XY gonads. Embryonic gonads at E11.5 were immunostained with antibodies against H3K9me2 (A) or H3K9me3 (B). Gonadal somatic cells were marked with anti-Gata4 antibodies. G, gonads; M, mesonephroi. Scale bar, 50 μm.
(PDF)

S2 Fig Representative FSC/SSC dot blot (left) and fluorescence histogram (right) showing gates used for sorting hCD271-nega, hCD271-low and hCD271-high cells from *Nr5a1-hCD271*-tg (XY) gonads and mesonephroi. (PDF)

S3 Fig ChIP analysis using purified gonadal somatic cells. (A) Schematic illustration of the purification of gonadal somatic cells for ChIP analysis. Two-cell embryos were prepared by *in vitro* fertilization using sperm derived from *Jmjd1a*^Δ/+^;*GLP* ^Δ/+^;*Nr5a1-hCD271*-tg males and oocytes derived from *Jmjd1a*^Δ/+^ females and were then transferred to pseudopregnant recipients. After in utero development, the embryos were collected at tail somite stages 17–19. After genotyping analysis, gonadal somatic cells were labeled with anti-hCD271 antibody and then purified through affinity columns. Cells corresponding to two to four embryos of each genotype were pooled and then subjected to ChIP analysis. (B) Numbers of purified gonadal somatic cells at tail somite stages 17–19 of the indicated genotypes. The numbers of gonadal somatic cells were consistent regardless of the genotypes. Numbers of examined embryos are shown above the bars.
(PDF)

S4 Fig Epigenetic states other than H3K9me2 of the *Sry* locus in gonadal somatic cells of E11.5 XY embryos. (A) Gonadal somatic cells of the indicated genotypes were purified according to the method described in S3 Fig, pooled for each genotype (2 to 4 embryos), and then subjected to ChIP-qPCR analyses for H3K4me3 (left) and H3K9ac (right). There was no significant difference of the modification levels between control and mutant gonads. (B) DNA methylation levels of the linear promoter region of *Sry* were quantified by bisulfite sequence analysis. hCD271-tagged gonadal somatic cells were fractionated into hCD271-high (Nr5a1-high) and hCD271-low (Nr5a1-low) populations as shown in S2 Fig. In control gonads, Sry-expressing cells were enriched predominantly in the hCD271-high population (Fig 1E). Analyzed CpG sequences of the *Sry* promoter region are presented at the top. The CpG positions are indicated relative to the start codon. (C) Summary of CpG methylation levels of the *Sry* promoter region. In a comparison of the DNA methylation levels in hCD271-high populations, we could not find significant levels for the difference between *Jmjd1a*^Δ/+^ and *Jmjd1a*^Δ/Δ^ littermates. *P* values were obtained using the Mann–Whitney *U*-test.
(PDF)

S5 Fig *The G9a* heterozygous mutation does not rescue the sex-reversal phenotype of XY Jmjd1a-deficient embryos. (A) Immunofluorescence analysis with antibodies against Sox9 and Foxl2 on E13.5 embryonic gonadal sections of the indicated genotypes. Scale bar, 50 μm. (B) Quantification of Sox9- and Foxl2-positive cells in E13.5 gonads. Numbers of examined embryos are shown above the bars. Data are presented as mean ± SD. ** *P* < 0.01; n.s., not significant.
(PDF)

S6 Fig *The GLP* heterozygous mutation induces reduction in the level of G9a protein. Gonads/mesonephroi of E11.5 XY embryos were stained with antibodies against GLP (A) and G9a (B), in combination with anti-Nr5a1 antibodies. (left) Representative data of flow-cytometric dot-blot analysis of the indicated proteins. (right) Plots of median fluorescence intensity (MFI) values for the indicated proteins in Nr5a1-positive gonadal somatic cells. *** *P* < 0.001.
(PDF)

S7 Fig Overexpression of *GLP* does not influence *Sry* expression levels in E11.5 XY gonads. We had previously established *GLP*-tg mice that carry an extra copy of *GLP* cDNA in the *Rosa26* locus [20]. In this line, the exogenous *GLP* cDNA is expressed ubiquitously by CAG promoter. Although *GLP* mRNA was actually overexpressed in the gonads of XY *GLP*-tg embryos at E11.5, mRNA levels of *Nr5a1*, *Sry* and *Sox9* were not affected.
(PDF)

S8 Fig Analysis of the sex development of XY *Jmjd1a*^Δ/Δ^; *Eset*^Δ/+^ embryos. (A) Comparison of *Eset* genomic sequences between wild-type and mutant alleles, generated by genome editing with the CRISPR/Cas9 system. We intended to disrupt the exon8 encoding TUDOR domain of *Eset*. Two guide RNAs (gRNAs), corresponding to a sequence within intron 7 and a sequence nearly at the 3’ end of exon 8, were introduced with *Cas9* mRNA into fertilized eggs of C57BL/6 mice. Dashes represent deleted sequences in the mutant allele. Capital and lower-case letters represent exonic and intronic sequences, respectively. (B) Genotyping for the *Eset* mutant allele by PCR. Location of the primers is indicated in (A). (C) Phenotype analysis of the *Eset*-mutant mice established in this study. No *Eset* homozygous mutant embryos were found among 34 embryos derived from the mating of *Eset* heterozygous mutant mice, indicating *Eset* homozygous mutant embryos died and were absorbed by E13.5. (D) Evaluation of the gonadal sex differentiation of E13.5 XY *Jmjd1a*^Δ/Δ^; *Eset*^Δ/+^ embryos by immunofluorescence analysis for Sox9 and Foxl2. (E) The ratios of Sox9- and Foxl2-positive cells of the indicated genotypes are summarized. *Eset* heterozygous mutation did not affect the sex development of Jmjd1a-deficient mice. Numbers of embryos examined are shown above the bars. Data are presented as mean ± SD. *** *P* < 0.001; n.s., not significant.
(PDF)
