# Supplementary material for: Rescuing the aberrant sex development of H3K9 demethylase Jmjd1a-deficient mice by modulating H3K9 methylation balance
Source: PLoS Genet. 2017 Sep 26;13(9):e1007034. doi: 10.1371/journal.pgen.1007034 (PMC5630185; doi:10.1371/journal.pgen.1007034)
Supplement: S1 Fig — Embryonic gonads at E11.5 were immunostained with antibodies against H3K9me2 (A) or H3K9me3 (B). Gonadal somatic cells were marked with anti-Gata4 antibodies. G, gonads; M, mesonephroi. Scale bar, 50 μm. (PDF) [file pgen.1007034.s001.pdf]

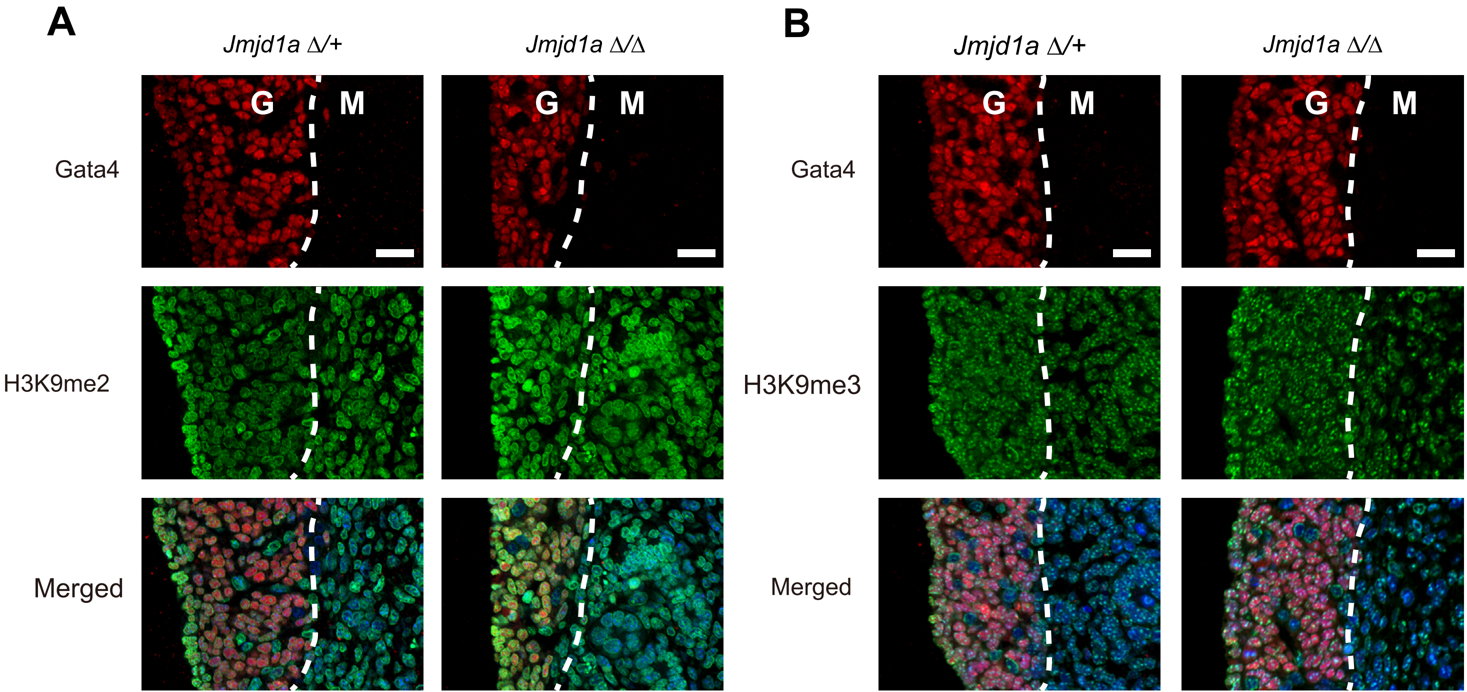
A
Jmjd1a Δ/+
Jmjd1a Δ/Δ
B
Jmjd1a Δ/+
Jmjd1a Δ/Δ
G
M
Gata4
H3K9me2
Merged
Gata4
H3K9me3
Merged
