# Supplementary figures and images for: Rescuing the aberrant sex development of H3K9 demethylase Jmjd1a-deficient mice by modulating H3K9 methylation balance
Source: PLoS Genet. 2017 Sep 26;13(9):e1007034. doi: 10.1371/journal.pgen.1007034 (PMC5630185; doi:10.1371/journal.pgen.1007034)

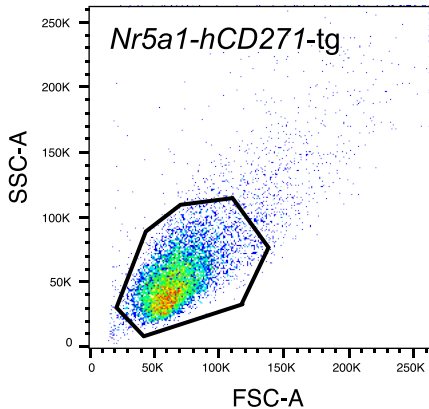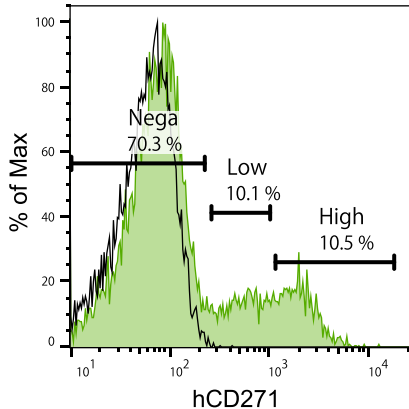

Supplement: S2 Fig — (PDF) [file pgen.1007034.s002.pdf]

**A**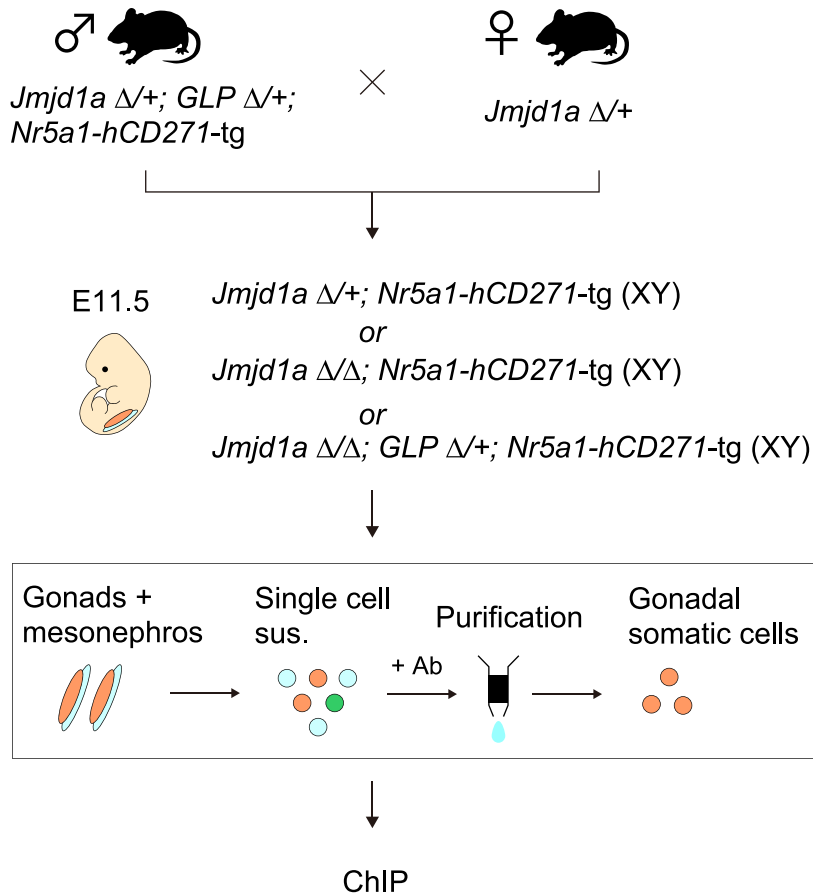**B**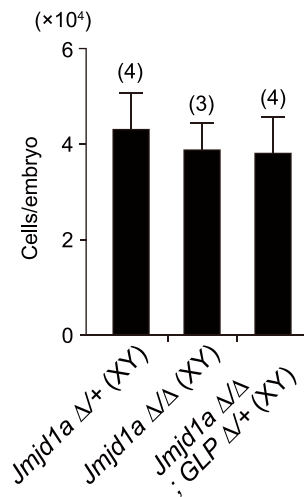

Supplement: S3 Fig — (A) Schematic illustration of the purification of gonadal somatic cells for ChIP analysis. Two-cell embryos were prepared by in vitro fertilization using sperm derived from Jmjd1aΔ/+;GLP Δ/+;Nr5a1-hCD271-tg males and oocytes derived from Jmjd1aΔ/+ females and were then transferred to pseudopregnant recipients. After in utero development, the embryos were collected at tail somite stages 17–19. After genotyping analysis, gonadal somatic cells were labeled with anti-hCD271 antibody and then purified through affinity columns. Cells corresponding to two to four embryos of each genotype were pooled and then subjected to ChIP analysis. (B) Numbers of purified gonadal somatic cells at tail somite stages 17–19 of the indicated genotypes. The numbers of gonadal somatic cells were consistent regardless of the genotypes. Numbers of examined embryos are shown above the bars. (PDF) [file pgen.1007034.s003.pdf]

**A**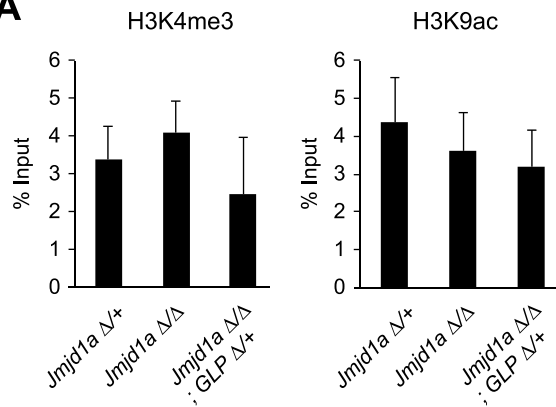**B**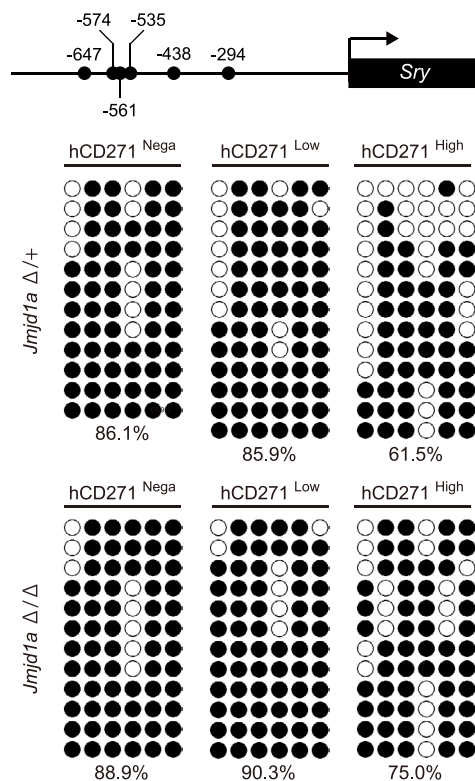**C**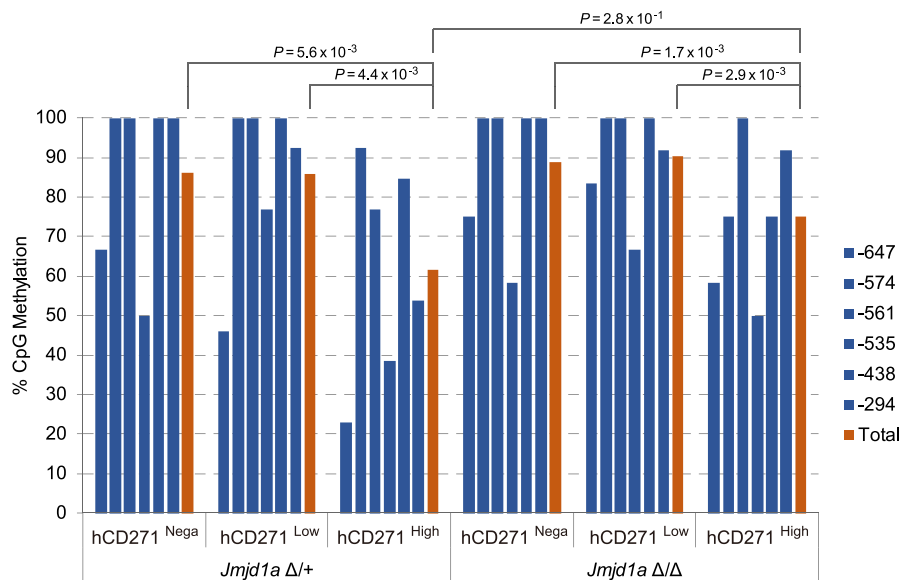

Supplement: S4 Fig — (A) Gonadal somatic cells of the indicated genotypes were purified according to the method described in S3 Fig, pooled for each genotype (2 to 4 embryos), and then subjected to ChIP-qPCR analyses for H3K4me3 (left) and H3K9ac (right). There was no significant difference of the modification levels between control and mutant gonads. (B) DNA methylation levels of the linear promoter region of Sry were quantified by bisulfite sequence analysis. hCD271-tagged gonadal somatic cells were fractionated into hCD271-high (Nr5a1-high) and hCD271-low (Nr5a1-low) populations as shown in S2 Fig. In control gonads, Sry-expressing cells were enriched predominantly in the hCD271-high population (Fig 1E). Analyzed CpG sequences of the Sry promoter region are presented at the top. The CpG positions are indicated relative to the start codon. (C) Summary of CpG methylation levels of the Sry promoter region. In a comparison of the DNA methylation levels in hCD271-high populations, we could not find significant levels for the difference between Jmjd1aΔ/+ and Jmjd1aΔ/Δ littermates. P values were obtained using the Mann–Whitney U-test. (PDF) [file pgen.1007034.s004.pdf]

**A**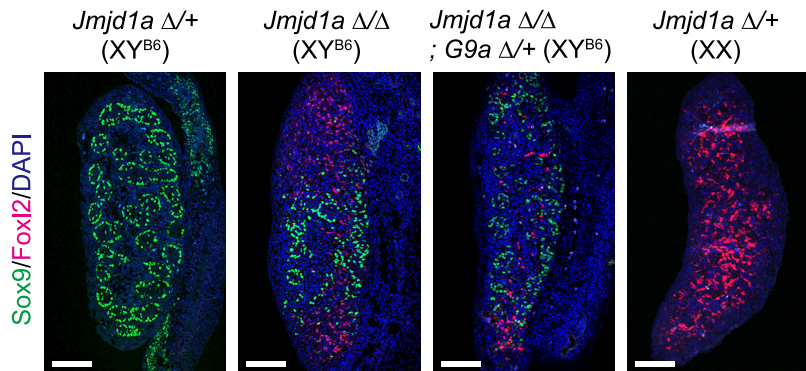**B**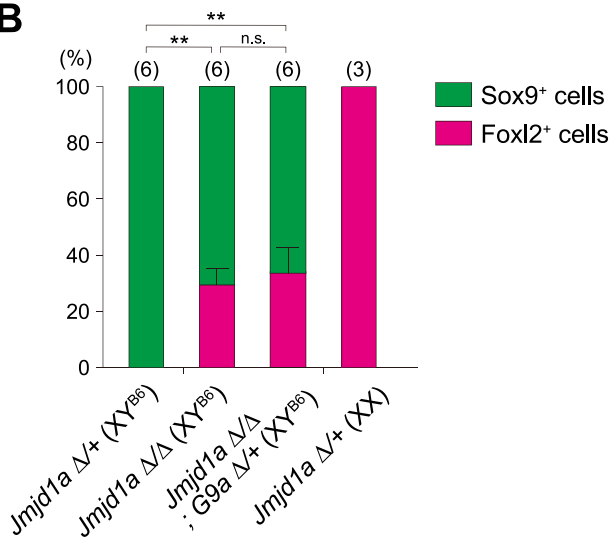

Supplement: S5 Fig — (A) Immunofluorescence analysis with antibodies against Sox9 and Foxl2 on E13.5 embryonic gonadal sections of the indicated genotypes. Scale bar, 50 μm. (B) Quantification of Sox9- and Foxl2-positive cells in E13.5 gonads. Numbers of examined embryos are shown above the bars. Data are presented as mean ± SD. ** P < 0.01; n.s., not significant. (PDF) [file pgen.1007034.s005.pdf]

**A**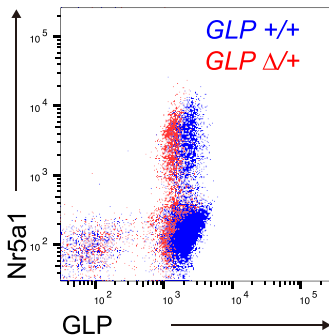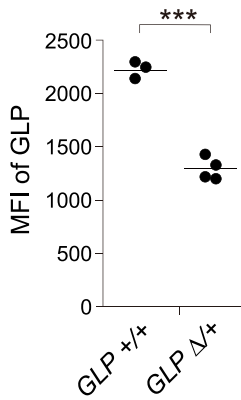**B**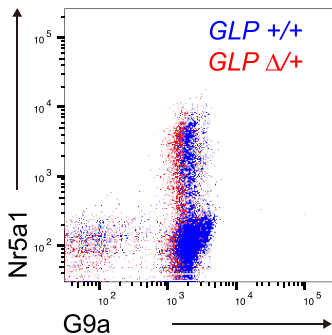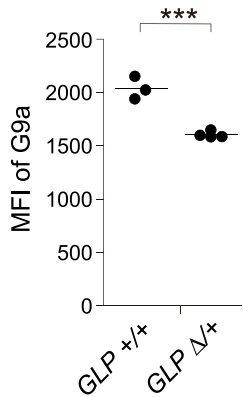

Supplement: S6 Fig — Gonads/mesonephroi of E11.5 XY embryos were stained with antibodies against GLP (A) and G9a (B), in combination with anti-Nr5a1 antibodies. (left) Representative data of flow-cytometric dot-blot analysis of the indicated proteins. (right) Plots of median fluorescence intensity (MFI) values for the indicated proteins in Nr5a1-positive gonadal somatic cells. *** P < 0.001. (PDF) [file pgen.1007034.s006.pdf]

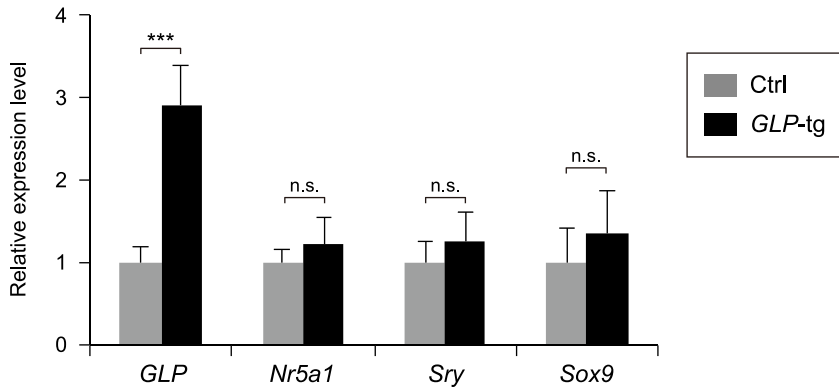

Supplement: S7 Fig — We had previously established GLP-tg mice that carry an extra copy of GLP cDNA in the Rosa26 locus [20]. In this line, the exogenous GLP cDNA is expressed ubiquitously by CAG promoter. Although GLP mRNA was actually overexpressed in the gonads of XY GLP-tg embryos at E11.5, mRNA levels of Nr5a1, Sry and Sox9 were not affected. (PDF) [file pgen.1007034.s007.pdf]

A

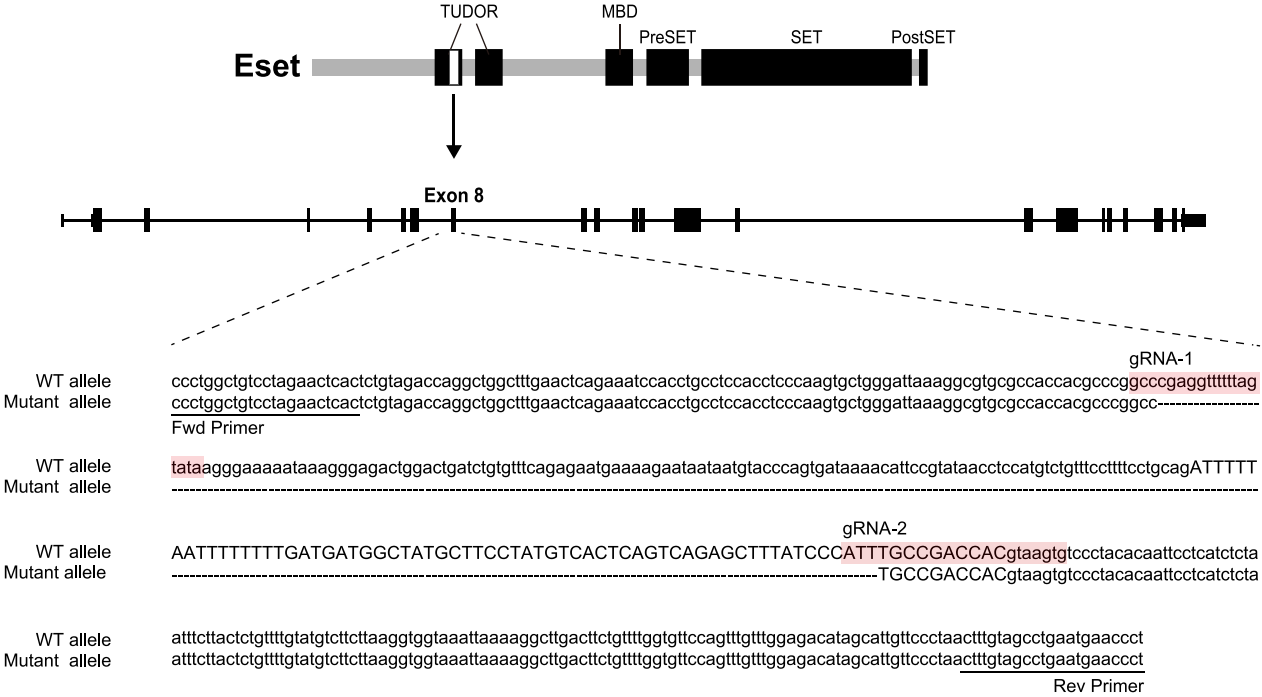

B

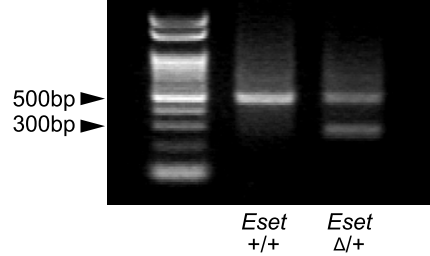

C

*Eset* Δ/+ (♀) × *Eset* Δ/+ (♂)

Number of Pups (E13.5)

| <i>Eset</i> +/+ | <i>Eset</i> Δ/+ | <i>Eset</i> Δ/Δ |
|-----------------|-----------------|-----------------|
| 32%             | 68%             | 0%              |
| (11/34)         | (23/34)         | (0/34)          |

D

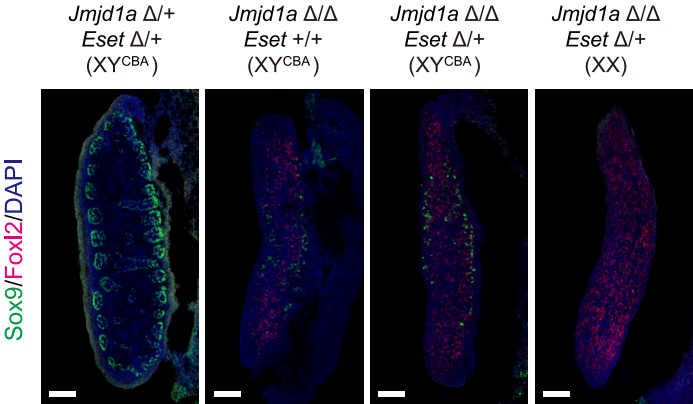

E

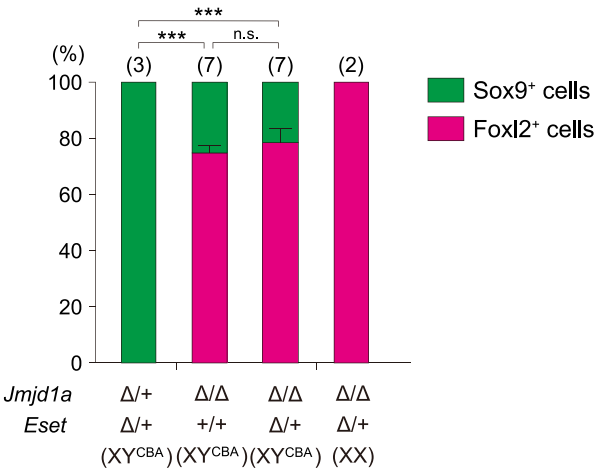

Supplement: S8 Fig — (A) Comparison of Eset genomic sequences between wild-type and mutant alleles, generated by genome editing with the CRISPR/Cas9 system. We intended to disrupt the exon8 encoding TUDOR domain of Eset. Two guide RNAs (gRNAs), corresponding to a sequence within intron 7 and a sequence nearly at the 3’ end of exon 8, were introduced with Cas9 mRNA into fertilized eggs of C57BL/6 mice. Dashes represent deleted sequences in the mutant allele. Capital and lower-case letters represent exonic and intronic sequences, respectively. (B) Genotyping for the Eset mutant allele by PCR. Location of the primers is indicated in (A). (C) Phenotype analysis of the Eset-mutant mice established in this study. No Eset homozygous mutant embryos were found among 34 embryos derived from the mating of Eset heterozygous mutant mice, indicating Eset homozygous mutant embryos died and were absorbed by E13.5. (D) Evaluation of the gonadal sex differentiation of E13.5 XY Jmjd1aΔ/Δ; EsetΔ/+ embryos by immunofluorescence analysis for Sox9 and Foxl2. (E) The ratios of Sox9- and Foxl2-positive cells of the indicated genotypes are summarized. Eset heterozygous mutation did not affect the sex development of Jmjd1a-deficient mice. Numbers of embryos examined are shown above the bars. Data are presented as mean ± SD. *** P < 0.001; n.s., not significant. (PDF) [file pgen.1007034.s008.pdf]
